# Septal and lateral wall localization of PBP5, the major D,D-carboxypeptidase of *Escherichia coli,* requires substrate recognition and membrane attachment

**DOI:** 10.1111/j.1365-2958.2010.07205.x

**Published:** 2010-06-07

**Authors:** Lakshmiprasad Potluri, Aneta Karczmarek, Jolanda Verheul, Andre Piette, Jean-Marc Wilkin, Nadine Werth, Manuel Banzhaf, Waldemar Vollmer, Kevin D Young, Martine Nguyen-Distèche, Tanneke den Blaauwen

**Affiliations:** 1 Department of Microbiology and Immunology, University of Arkansas for Medical SciencesLittle Rock, AR 72205-7199, US; 2 Molecular Cytology, Swammerdam Institute for Life Sciences, University of AmsterdamScience Park 904, 1098 XH Amsterdam, P.O. Box 94215, 1090 GE Amsterdam, the Netherlands; 3 Centre d'Ingénierie des Protéines, Institut de Chimie B6a, Université de LiègeB-4000 Sart Tilman, Belgium; 4 Mikrobielle Genetik, Eberhard Karls Universität TübingenTübingen, Germany; 5 Centre for Bacterial Cell Biology, Newcastle University, Framlington PlaceNewcastle upon Tyne, NE2 4HH, UK

## Abstract

The distribution of PBP5, the major D,D-carboxypeptidase in *Escherichia coli*, was mapped by immunolabelling and by visualization of GFP fusion proteins in wild-type cells and in mutants lacking one or more D,D-carboxypeptidases. In addition to being scattered around the lateral envelope, PBP5 was also concentrated at nascent division sites prior to visible constriction. Inhibiting PBP2 activity (which eliminates wall elongation) shifted PBP5 to midcell, whereas inhibiting PBP3 (which aborts divisome invagination) led to the creation of PBP5 rings at positions of preseptal wall formation, implying that PBP5 localizes to areas of ongoing peptidoglycan synthesis. A PBP5(S44G) active site mutant was more evenly dispersed, indicating that localization required enzyme activity and the availability of pentapeptide substrates. Both the membrane bound and soluble forms of PBP5 converted pentapeptides to tetrapeptides *in vitro* and *in vivo*, and the enzymes accepted the same range of substrates, including sacculi, Lipid II, muropeptides and artificial substrates. However, only the membrane-bound form localized to the developing septum and restored wild-type rod morphology to shape defective mutants, suggesting that the two events are related. The results indicate that PBP5 localization to sites of ongoing peptidoglycan synthesis is substrate dependent and requires membrane attachment.

## Introduction

The morphology of most bacteria is determined by the interplay of cytoskeleton-like proteins with enzymes that fabricate the peptidoglycan cell wall, although the mechanisms by which any one cell shape is created and maintained remain largely undefined (Den Blaauwen *et al*., 2008; Vollmer and Bertsche, 2008). The peptidoglycan layer of *Escherichia coli* (*E. coli*) is a covalently closed network of sugar strands that are interconnected by peptide side bridges. The peptidoglycan-building unit, the disaccharide pentapeptide *N*-acetyl-glucosamine-β-1,4-*N*-acetyl-muramyl pentapeptide, is inserted into the existing peptidoglycan layer by the combined synthetic and hydrolytic activities of the penicillin-binding proteins (PBPs). The hydrolytic activity of PBPs is necessary for cell growth, because the covalent bonds of the peptidoglycan layer have to be broken for the insertion of new subunits (Vollmer and Bertsche, 2008). The pentapeptide consists of L-Ala-*γ-*D-Glu-*m*-A_2_pm-D-Ala-D-Ala, where *m*-A_2_pm stands for *meso*-diaminopimelic acid. Peptidoglycan polymer synthesis is catalysed by high molecular mass PBPs, of which the Class A types possess both transglycosylase and transpeptidase activities that extend the glycan chains and cross-link the peptide side bridges (PBP1A, PBP1B and PBP1C), and the Class B types possess only transpeptidase activity (PBP2 and PBP3) (Sauvage *et al*., 2008). In the transpeptidase reaction, the amino group of *m*-A_2_pm and the carbonyl group of D-Ala residues of adjacent glycan strands are coupled to form a peptide cross bridge, while concomitantly releasing the terminal D-Ala from the donor peptide strand. Low molecular mass Class C PBPs have either D,D-carboxypeptidase activity (PBP5, PBP6 and PBP6B/DacD), which removes the terminal D-Ala of the pentapeptide chain in a reaction similar to transpeptidation, or else they have endopeptidase activity (PBP4 and PBP7), which cleaves the peptide cross bridges (Ghosh *et al*., 2008).

The structure of the most abundant D,D-carboxypeptidase PBP5 (Fig. S1) consists of two modules oriented ∼90° to one another (Davies *et al*., 2001; Nicholas *et al*., 2003). Module I (amino acids 1–262) contains the D,D-carboxypeptidase active site, which is situated at the interface of a five-stranded antiparallel β-sheet packed on both sides by α helices. Module II (amino acids 263–374) consists of two antiparallel β-sheets plus an amphipathic carboxy-terminal helix, with this latter being responsible for attaching PBP5 to the cytoplasmic membrane (Pratt *et al*., 1986; Phoenix and Pratt, 1990). (Note that the amino acid numbering for PBP5 does not include the original residues 1–29, which represent the signal sequence that is cleaved once the protein is translocated to the periplasm.)

*E. coli* mutants lacking PBP5 show a variety of morphological defects such as branches, bends and kinks (Young, 2003). Deletion of the other D,D-carboxypeptidases enhances these morphological aberrations, which can subsequently be corrected by the expression of PBP5 (Nelson and Young, 2001). The gene for the 41.6 kDa protein PBP5 (*dacA*) is part of an operon that also contains the genes coding for PBP2 and RodA (*pbpA/mrdA* and *rodA/mrdB* respectively) (Stoker *et al*., 1983), proteins that are both essential for cell elongation. Overproduction of PBP5 in normal rod-shaped cells results in spherical growth (Markiewicz *et al*., 1982) and has been claimed to restore cell division at the non-permissive temperature in cells carrying the *ftsI23* allele, which produces a temperature sensitive version of PBP3 (Begg *et al*., 1990). Curiously, the temperature sensitive phenotype of the missense mutations *ftsK44* and *ftsK3531* are suppressed by deleting the *dacA* gene (Begg *et al*., 1995; Draper *et al*., 1998). These observations suggest that PBP5 may play a role in balancing the growth of cell length versus septal peptidoglycan synthesis. This putative regulatory role is not exerted by a fluctuating concentration of PBP5 during the cell cycle, which appears to be constant (Wientjes *et al*., 1983), hovering near 317 ± 69 molecules per cell when grown in M9 minimal medium and 790 ± 105 molecules per cell when grown in rich medium (Dougherty *et al*., 1996). An alternate possibility is that the cellular localization of PBP5 varies over the cell cycle and helps to regulate peptidoglycan synthesis.

We analysed the localization of endogenous PBP5 in mutants defective in elongation or cell division, by using immunolocalization and by visualizing GFP-PBP5 fusion proteins in live cells. PBP5 localized in the cylindrical envelope as well as at the division site, and the amount of PBP5 at these sites correlates with the location and intensity of peptidoglycan synthesis. These localization patterns were lost when the active site serine of PBP5 was mutated or when the membrane-associated, carboxy-terminal amphipathic helix was removed. We conclude, first, that PBP5 localization depends on the continued synthesis of peptidoglycan instead of requiring direct interactions with proteins comprising the peptidoglycan-polymerizing machinery, and second, that the morphological function of periplasmic PBP5 requires a defined interaction between PBP5 and the inner membrane that enables the protein to localize properly at the division septum.

## Results

### PBP5 localizes in the lateral envelope and at septal constrictions

PBP5 affinity-purified antiserum recognized PBP5 as well as PBP6 on immunoblots and showed an envelope localization pattern in *dacA* deletion strains, which are devoid of PBP5 (data not shown). These results indicated that at least some of the antibodies were cross-reactive. To eliminate these antibodies, the affinity-purified antiserum was incubated with a cell extract derived from the *dacA* deletion strain *E. coli* CS12-7. This step removed cross-reactive antibodies against PBP6 and allowed antibodies against PBP5 to remain in the supernatant. The procedure yielded an IgG fraction that recognized a single band on an immunoblot with a mass that corresponded to that of PBP5 (Fig. S2D) and that showed no staining in the *dacA* deletion strain CS12-7 (Fig. S2B) or in strain CS703-1 (Fig. S2C), which lacks PBP 1A, 4, 5, 6 and 7, as well as the AmpC β-lactamase and the AmpH β-lactamase-like protein (Denome *et al*., 1999). This latter strain grows with a severely aberrant morphology (Denome *et al*., 1999; Meberg *et al*., 2001) that is complemented by the expression of active PBP5 (Nelson and Young, 2000; 2001;). In the parental strain, *E. coli* CS109, PBP5 localized as foci in the lateral wall, with very few foci at the old cell poles but somewhat more pronounced at midcell (Fig. S2A and E). This IgG fraction was used for all further studies of the localization pattern of PBP5.

### An sfGFP-PBP5 fusion protein is functional and localizes to division sites

**Fig. 1:**
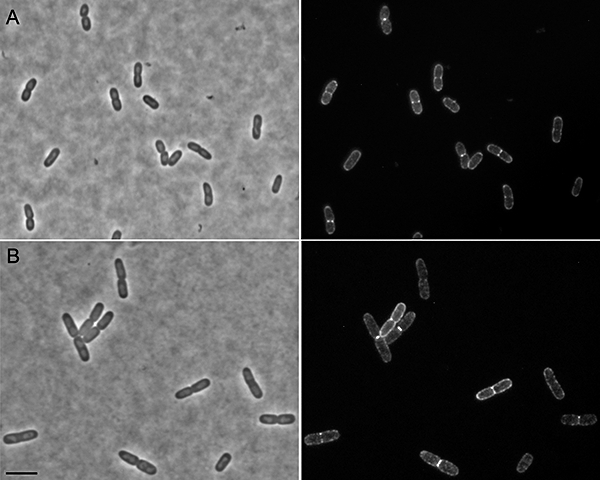
sfGFP-PBP5 is a functional fusion and localizes to active peptidoglycan-synthesizing regions. A. sfGFP-PBP5 complements the cell shape defects of *E. coli* CS703-1, which lacks the three major D,D-carboxypeptidases, and localizes to the cell periphery and to septa of constricting cells. B. A Similar localization pattern was observed in the parent strain, CS109. Phase contrast images are on the left and fluorescence images are on the right. All images have the same magnification, and the scale bar in panel B equals 5 µm.

To confirm the immunolocalization pattern of PBP5 in living cells, we fused green fluorescent protein (GFP) to PBP5 so that the fusion protein would be transported to the periplasm. In Gram-negative bacteria, when wild-type GFP is exported to the periplasm via the general secretion (Sec) pathway the protein does not fold correctly and does not become fluorescent (Feilmeier *et al*., 2000). However, previous work showed that pre-folded GFP and proteins fused to GFP were exported to the periplasm via the twin-arginine transport pathway (Bernhardt and de Boer, 2003; 2004;). With this expectation, we created a TorA-GFP-PBP5 fusion protein in an attempt to determine the localization of fluorescently tagged PBP5 in live cells. Unfortunately, this GFP-PBP5 protein was not exported to the periplasm, for reasons that are under investigation (L. Potluri, H. Lavender and K.D. Young, unpublished). To circumvent this problem, we fused PBP5 to a newly created enhanced folding variant of GFP, superfolder GFP (sfGFP), which is exported by the SRP pathway and folds properly to become fluorescent in the periplasm (Uehara *et al*., 2009). We constructed a *dsbA(SS)-sfgfp*-*dacA* gene that encodes an sfGFP-PBP5 fusion protein with the signal sequence of DsbA at its amino terminus. To determine if this protein was exported to the periplasm, we placed the gene under control of the *lac* promoter in a medium copy plasmid, pLP4, and expressed the protein in *E. coli* CS703-1. The DsbA(SS)-sfGFP-PBP5 protein was highly fluorescent and complemented the shape defects of CS703-1 so that the cells grew with virtually normal rod-shaped morphology and the PBP5 fusion protein was distributed around the cell periphery, appearing as a thin fluorescent line studded with numerous bright spots (Fig. 1A). Cells in the process of division (constricting cells) also exhibited bright rings or spots at the septa (Fig. 1A). In addition, a faint fluorescent band appeared at the centre of some of the elongated cells that had not yet formed a visible constriction (Fig. 1). The same localization pattern was observed when DsbA(SS)-sfGFP-PBP5 was expressed in the parental strain CS109 (Fig. 1B). These localization patterns paralleled the distribution of PBP5 as determined by immunofluorescence (see Fig. S2 and below).

### PBP5 localization at the division site requires the presence of FtsZ

**Fig. 2:**
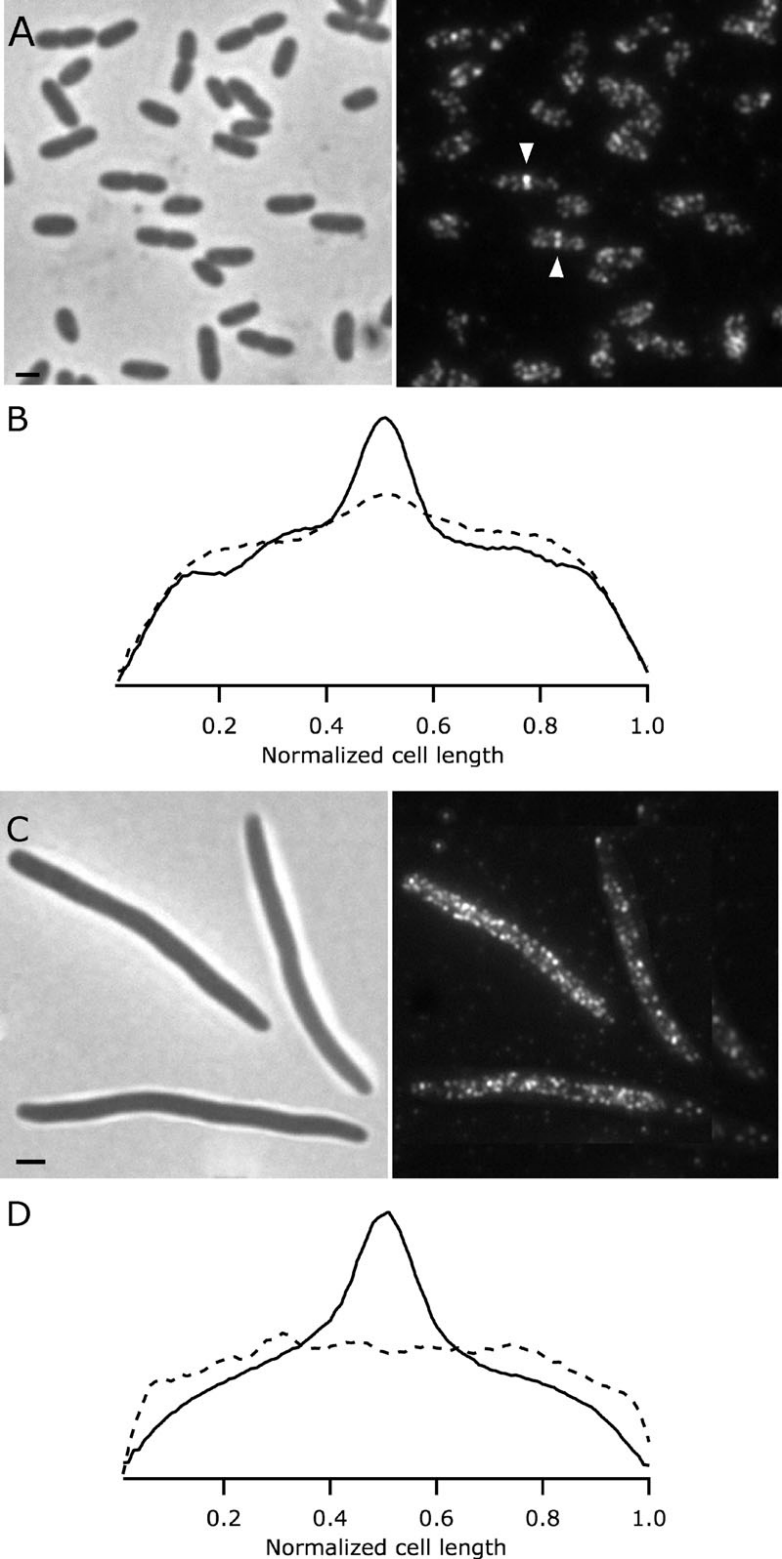
PBP5 localizes in the lateral cell envelope and at the division site in the presence of active FtsZ. A. Immunolocalization pattern of PBP5 in the wild-type strain LMC500, which is the parental strain of all temperature-sensitive strains used in this study. Phase contrast images are on the left and fluorescence images are on the right. Arrows indicate midcell localization. The bar equals 1 µm. B. Average fluorescence profiles per cell plotted against their normalized cell length. The dashed profile is from non-constricting cells (*n* = 360) and the solid line from constricting cells (*n* = 140). C. Immunolocalization pattern of PBP5 in the FtsZ84(ts) strain LMC509 at the restrictive temperature. Phase contrast images are on the left and fluorescence images are on the right. The bar equals 1 µm. D. Average fluorescence profiles per cell plotted against normalized cell length. The dashed profile is from filaments after a shift for two MDs to 42°C (*n* = 150) and the solid line from cells grown to steady state at the permissive temperature (*n* = 500). Note that PBP5 does not localize preferentially at midcell in the absence of localized FtsZ.

The formation of the FtsZ ring at the middle of the cell is the earliest known step of cell division. Once the FtsZ ring is formed (Den Blaauwen *et al*., 1999), after about one-fifth of a generation time, other proteins are recruited (Aarsman *et al*., 2005) and together they form the multi-protein divisome complex (Nanninga, 1991). The age at which a protein starts to localize at midcell can be calculated from the fraction of cells that show protein midcell localization in steady state grown cells (Den Blaauwen *et al*., 1999). The timing of divisome maturation is well established in *E. coli* LMC500, so we visualized PBP5 by immunostaining fixed cells of this strain after growing to steady state in GB1 medium at 28°C. PBP5 localized in a spot-like pattern in the lateral envelope and more densely at the division site of constricting cells (Fig. 2A). The average fluorescence signal along the normalized cell length of about 140 cells showed a clear increase in the fluorescence signal at midcell of constricting cells (Fig. 2B). The increase in intensity at midcell was not a result of membrane constriction, i.e. merely due to a double layer of membrane [compare the fluorescence intensity profile of membranes labelled with the general lipid membrane stain, BODIPY-C12, in Fig. 8A of Bertsche *et al*. (Bertsche *et al.*, 2006)].

**Fig. 8:**
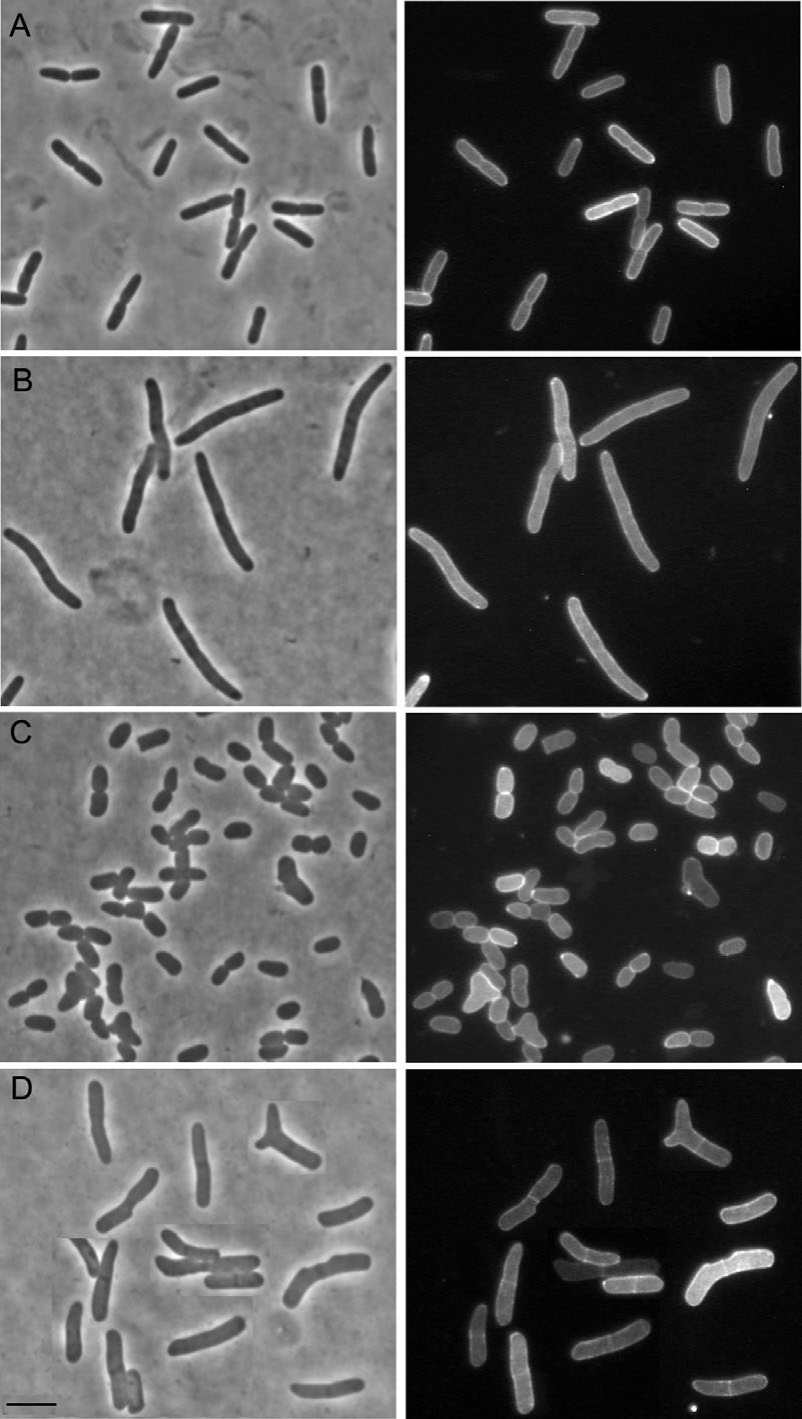
Septal localization of PBP5 requires membrane anchoring. Cells were grown in LB at 30°C in the presence or absence of 1 µg ml^−1^ aztreonam for one MD to inactivate PBP3. A. wild-type *E. coli* CS109 expressing the DsbA(SS)-sfGFP-PBP5CΔ18 fusion protein from plasmid pLP523. B. CS109 expressing the DsbA(SS)-sfGFP-PBP5CΔ18 fusion protein in the presence of aztreonam. C. CS703-1 expressing the DsbA(SS)-sfGFP-PBP5CΔ18 fusion protein from plasmid pLP523. D. CS703-1 expressing DsbA(SS)-sfGFP-PBP5CΔ18 fusion protein in the presence of aztreonam. The left side of each dual panel is the phase contrast image and the right side is the fluorescence image. Panel D contains a mosaic of images taken from different fields of view. All images have same magnification, and the scale bar in panel D equals 5 µm.

To determine whether midcell localization of PBP5 depended on specific divisome proteins, we visualized PBP5 in *E. coli* LMC509, which expresses the FtsZ84(ts) protein. This temperature-sensitive FtsZ variant does not polymerize when cells are grown at the restrictive temperature of 42°C. As a result, division is not initiated and the cells grow into filaments (Addinall *et al*., 1997). At 28°C, PBP5 localized in the same fashion as observed in the parent strain LMC500 (Fig. 2D). However, at 42°C, PBP5 was evenly distributed in the cell envelope with no pronounced presence at midcell (Fig. 2C and D). A similar distribution pattern was observed for the DsbA(SS)-sfGFP-PBP5 fusion protein (not shown). These results indicate that the septal localization of PBP5 depends directly or indirectly on the presence of FtsZ rings.

### Localization of PBP5 does not require a mature divisome

**Fig. 3:**
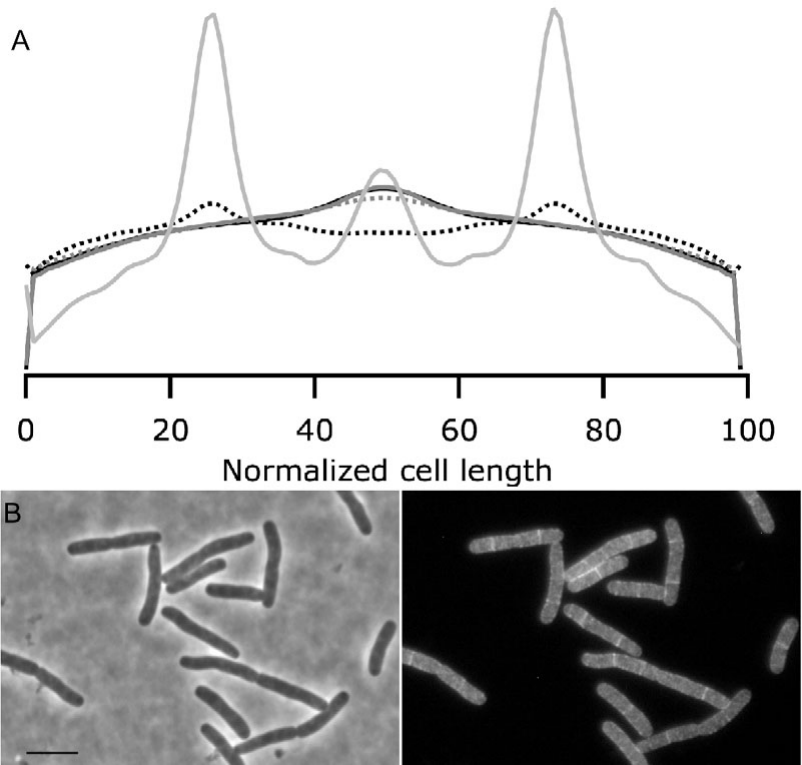
PBP5 localizes in FtsA(ts) filaments at the same positions as FtsZ localizes. A. Average fluorescence profiles per cell plotted against their normalized cell length. The surface below the graphs has been normalized to 1. wild-type LMC500 cells (dark grey lines) or LMC512 FtsA(ts) cells (black lines) grown to steady state in GB1 medium at the permissive temperature of 28°C (solid line) and after two MD at 42°C (dotted line) were immunolabelled with affinity-purified antibodies against PBP5. The light grey profile is from LMC512 cells grown for two MDs at the restrictive temperature and immunolabelled with antibodies against FtsZ. More than 1000 cells were analysed for each profile. B. Immunolocalization pattern of PBP5 in the Strain PS236 (ftsA12) expressing DsbA(SS)-sfGFP-PBP5 at restrictive temperature (42°C) for approximately two MDs. Cells were immediately fixed after harvest. Phase contrast images are on the left and fluorescence images are on the right. The bar equals 5 µm.

To determine whether PBP5 localization depended on one or more divisome proteins in addition to FtsZ, we visualized the distribution of PBP5 in filamentous FtsA(ts) cells. FtsA is an essential cell division protein and its presence is required for the recruitment of the late localizing divisome proteins FtsK upto FtsN (Wang and Lutkenhaus, 1998; Chen and Beckwith, 2001). The strain LMC512 that harbours a temperature-sensitive FtsA mutation (Taschner *et al*., 1988) and its parental strain LMC500 were grown to steady state in GB1 medium at 28°C and shifted for two mass doublings (MDs) to the restrictive temperature of 42°C. The average fluorescence profiles of the immunolabelled LMC500 and LMC512 strains showed a clear lateral wall and midcell localization of PBP5 at the permissive temperatures (Fig. 3A). However, at non-permissive temperatures PBP5 localization shifted to one quarter and three quarter positions in the FtsA(ts) filaments. This localization pattern of PBP5 coincided with the FtsZ localization (Fig. 3A light grey profile). A similar distribution pattern was observed for the DsbA(SS)-sfGFP-PBP5 fusion protein (Fig. 3B). These results suggest that septal localization of PBP5 does not require mature divisome.

To substantiate these results, PBP5 midcell localization was investigated in other cell division mutants. PBP3 is required for septal peptidoglycan synthesis and it localizes to the division site during the late stage of divisome assembly, and remains associated with the divisome until invagination and septation are completed (Weiss *et al*., 1999; Aarsman *et al*., 2005). *E. coli* LMC510 contains the *ftsI2158* mutation and expresses a temperature-sensitive variant of PBP3 that is not able to localize at the restrictive temperature (Bertsche *et al*., 2006). After growing the strain at 42°C for two MDs, LMC510 produced cell filaments with blunt constrictions where septation would normally occur.

**Fig. 4:**
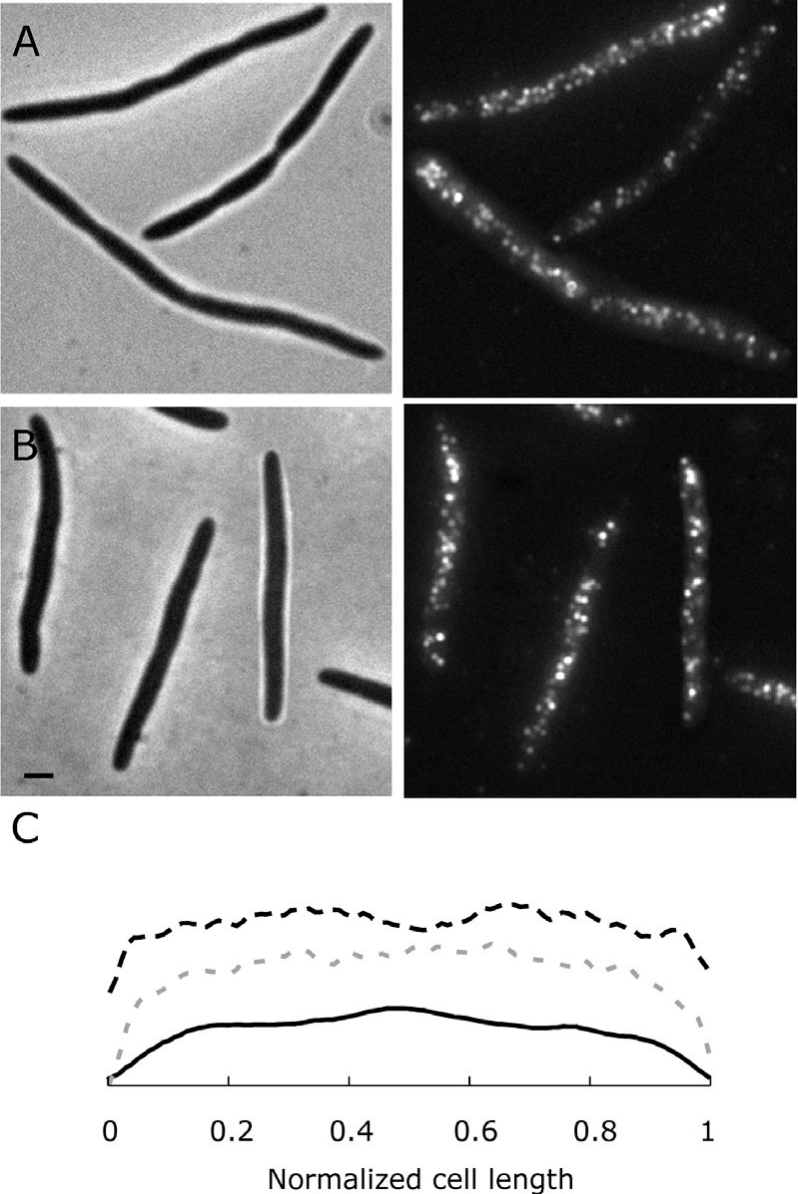
Immunolocalization does not detect septal localization of PBP5 when PBP3 is inactivated. A. Immunolocalization pattern of PBP5 in the FtsI2158(ts) strain LMC510 after growth in GB1 at the restrictive temperature (42°C) for two MDs. B. Immunolocalization pattern of PBP5 in the wild-type strain LMC500 after growth in GB1 at 28°C in the presence of 1 µg ml^−1^ aztreonam for two MDs. The bar equals 1 µm. C. Average fluorescence intensity profiles per average cell plotted against their normalized cell length. The profile of the black dashed line is from filaments after growth for two MDs in the presence of aztreonam (*n* = 154), the profile of the grey dashed line is from filaments grown after a shift for two MDs to 42°C (*n* = 165) and the profile of the solid line is from LMC510 cells grown to steady state at the permissive temperature (*n* = 486). The fluorescent profiles have been shifted with respect to each other to allow visualization of the shape of the profiles, which would have overlapped too much if their surface would have be normalized to 1.

When we utilized the immunolocalization procedure to locate PBP5 in filaments produced by inactivating PBP3(ts), the protein was not observed at midcell or at other potential division sites (Fig. 4A and C), implying that the positioning of PBP5 depended on the presence of PBP3 or, in conflict with the conclusion based on the FtsA(ts) results, possibly on a mature divisome. We also visualized the distribution of PBP5 in *E. coli* LMC500 in the presence of aztreonam, which inhibits the activity of PBP3 (Sykes and Bonner, 1985) but which allows PBP3 to remain at midcell for at least two MDs, after which a few percent of the cells develop divisomes at the one-quarter and three-quarter positions of the filaments (Den Blaauwen *et al*., 2003). If PBP5 localization depends on the presence of PBP3 but not on its activity, then PBP5 should localize to midcell even though cell division could not be completed. Instead, as measured by the immunolocalization procedure, PBP5 did not localize at midcell in aztreonam-induced filaments after two MDs but appeared to be distributed randomly around the cell envelope (Fig. 4B and C). At first, this pattern suggested that PBP5 localization needed an active PBP3 protein in the divisome. However, additional immunolabelling experiments showed that aztreonam-treated cells did have PBP5 rings when PBP5 was expressed ectopically from a plasmid (see below).

**Fig. 5:**
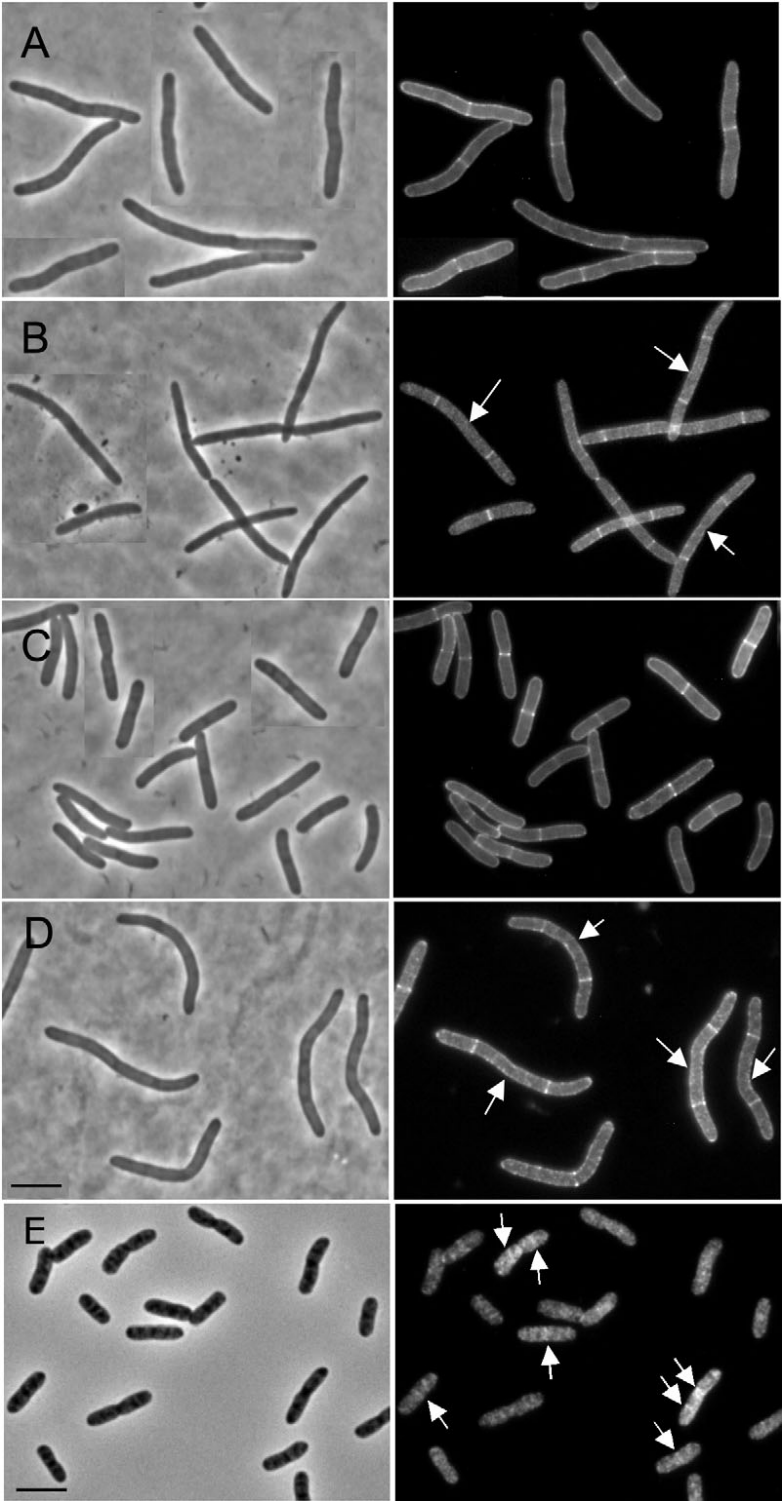
DsbA(SS)-sfGFP-PBP5 localizes to newly forming septa in the absence of active PBP3. A. The parent strain CS109 expressing DsbA(SS)-sfGFP-PBP5 after inactivation of PBP3 by exposure to 1 µg ml^−1^ aztreonam for one MD. B. Strain LP10-1 expressing DsbA(SS)-sfGFP-PBP5 at restrictive temperature (42°C) for approximately 1.5 MDs. Cells were immediately fixed after harvest. C. Strain CS703-1 expressing DsbA(SS)-sfGFP-PBP5 in the presence of aztreonam for one MD. D. Strain CS703-1 expressing DsbA(SS)-sfGFP-PBP5 in the presence of aztreonam for two MDs. Arrows (in panel B and D) indicate older aborted division sites. E. Strain CS703-1 expressing PBP5 in the presence of aztreonam for one MD and immunolabelled with antibodies against PBP5. The arrows (in panel E) indicate PBP5 localization at midcell, at one-quarter or at three-quarter positions of the cell. Phase contrast images are on the left and fluorescence images are on the right. The bars equal 5 µm.

In the preceding experiments, endogenous PBP5 was localized in fixed cells by using antibodies to detect the protein. However, there are cases in which the fixation protocol for immunolabelling alters or destroys the localization patterns of divisome-related proteins such as FtsZ, SufI/FtsP and AmiC (Bernhardt and de Boer, 2003; Peters *et al*., 2007; Tarry *et al*., 2009). Thus, the above procedure may not be as sensitive a localization method as one based on visualizing the fluorescence produced by proteins fused to GFP in living cells. Therefore, to determine if PBP5 localization did indeed depend on a relatively mature divisome, we followed the distribution of a DsbA(SS)-sfGFP-PBP5 fusion protein as it was expressed in aztreonam-induced cell filaments. In contrast to the immunolabelling results, sfGFP-PBP5 localized as a ring at midcell in aztreonam-induced cell filaments grown for one MD. After two MDs many filaments lost this midcell PBP5 ring, but in these cases two additional PBP5 rings appeared where nascent division sites were positioned at one quarter and at three quarters of the cell length (Fig. 5). To further confirm these observations, we visualized the distribution of DsbA(SS)-sfGFP-PBP5 in *E. coli* LMC510 (*ftsI*2158) and in *E. coli* LP10-1 (*ftsI*23), both of which express temperature sensitive versions of PBP3, and in *E. coli* LMC531 (*ftsQ*1), which expresses a temperature-sensitive FtsQ protein (van den Ent *et al*., 2008). Each strain was grown in GB1 and in TY medium at the permissive temperature of 28°C in the presence of 50 mM IPTG for 1 MD (to induce expression of the sfGFP-PBP5 protein) and then shifted to the restrictive temperature of 42°C, for another one or two MDs (to inhibit divisome assembly at different points). In all cases, PBP5 localized as a thin ring at midcell or at the one-quarter and three-quarter positions in longer cell filaments (Fig. 5B and Fig. S3). To address the differences between the results obtained by using the sfGFP-PBP5 fusion protein versus immunolabelling, we repeated the immunolabelling experiment with strain CS703-1, which is rich in pentapeptides, but PBP5 was expressed from the plasmid pLP515 and PBP3 activity was inhibited by growing cells in the presence of aztreonam for one MD. When these cells were immunolabelled with antibodies against PBP5, in almost all cells PBP5 localized at midcell or at the one-quarter and three-quarter positions of the cell (Fig. 5E). Thus, these results showed that localization of PBP5 to the septal ring required the presence of FtsZ but did not require the presence of proteins that arrive later in divisome assembly (i.e. FtsK, FtsQ, FtsW, FtsI/PBP3, PBP1B, FtsN and AmiC).

The difference in results obtained by immunolabelling of endogenous PBP5 versus those obtained by observing the GFP-PBP5 fusion protein could be due to the loss of protein during fixation prior to immunolabelling. However, when PBP5 was expressed from plasmid (Fig. 5E) or when we fixed and permeabilized aztreonam-induced cell filaments of *E. coli* CS703-1, the DsbA(SS)-sfGFP-PBP5 protein was still visible at midcell (Fig. 5E and data not shown), suggesting that the fixation procedure did not cause a significant loss of PBP5. We note that GFP-PBP5 produced images in which the fluorescence was distributed smoothly within the cell periplasm, whereas immunolabelling with primary and secondary antibodies produced a coarser, granular pattern. Because the number of native PBP5 molecules is 300–700 per cell depending on the growth rate (Dougherty *et al*., 1996), we believe the very thin rings of endogenous PBP5 in cells and cell filaments were simply too subtle to be detected by routine immunolabelling. Consistent with this supposition, in CS703-1, a pentapeptide-rich strain, we detected PBP5 rings by immunolabelling when the protein was expressed ectopically from a plasmid. These results suggest that detecting PBP5 rings with this technique requires a higher concentration of PBP5 and supports the observations of sfGFP-PBP5 localization patterns.

### PBP5 localization depends on its active site

**Fig. 6:**
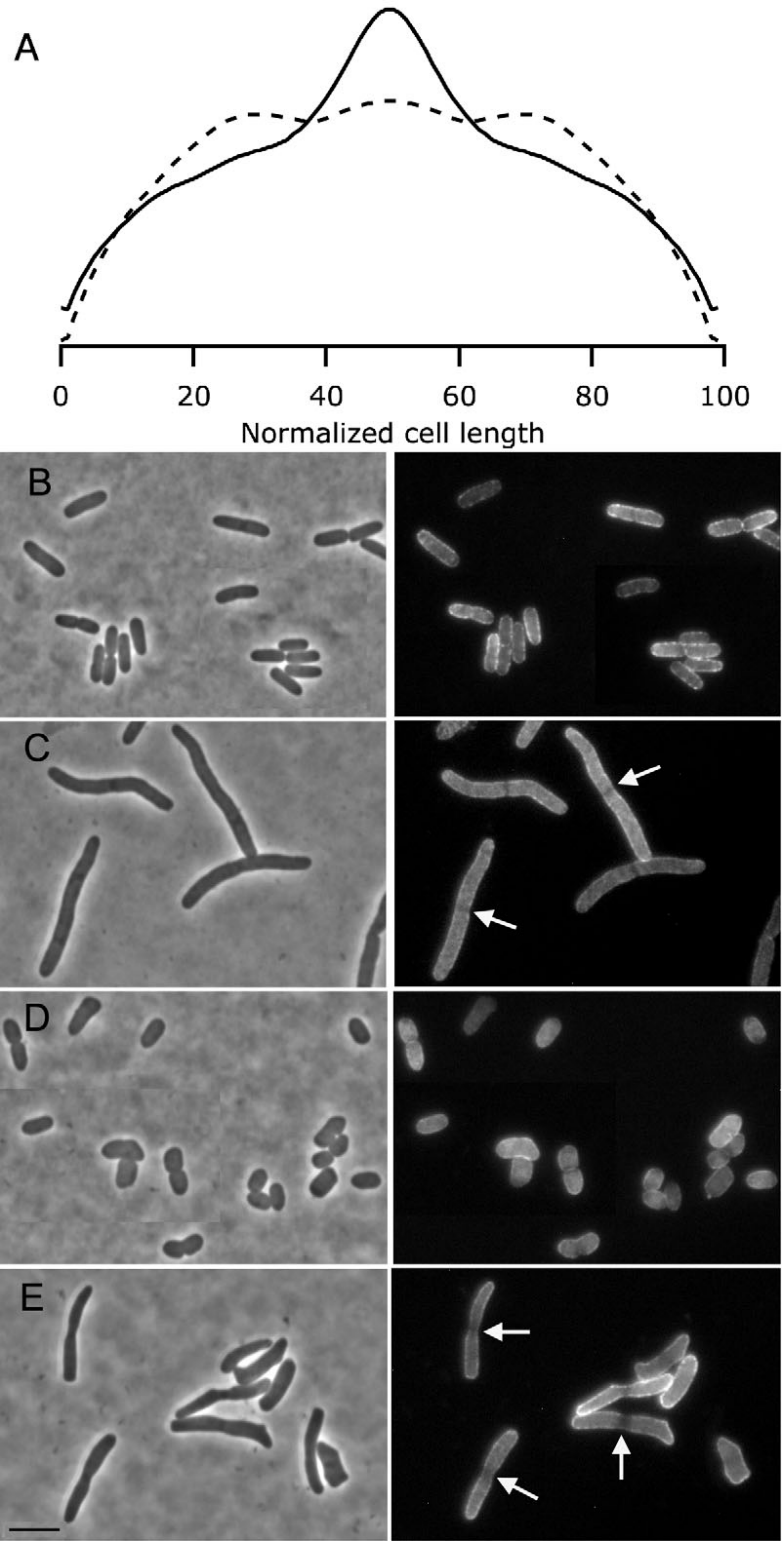
The PBP5(S44G) active site mutant protein localizes more evenly in the envelope than the PBP5(wt) protein. Cells were grown in LB at 30°C for one MD in the presence or absence of 1 µg ml^−1^ aztreonam. A. Average fluorescence intensity profiles plotted against the normalized cell length of CS703-1 cells expressing wild-type PBP5 (from pLP515, solid profile) and CS703-1 cells expressing PBP5(S44G) (from pLP514, dashed profile). For each profile approximately 1000 cells were measured. Representative photographs are shown for the parent strain CS109 expressing DsbA(SS)-sfGFP-PBP5(S44G) in the absence (B) or presence (C) of aztreonam, and for *E. coli* CS703-1 expressing DsbA(SS)-sfGFP PBP5(S44G) in the absence (D) and presence (E) of aztreonam. Note that the PBP5(S44G) active site mutant seldom localizes to septal sites (shown with arrows), compared with the localization of wild-type PBP5 (Fig. 5). Phase contrast images are on the left and fluorescence images are on the right. The bar equals 5 µm.

The affinity of PBP5 for division sites could be because it interacts with one or more divisome proteins or because it interacts with its pentapeptide substrate, which is concentrated at the septum during cell division. If the interaction were substrate-dependent, then mutating the active site of PBP5 should reduce its affinity for pentapeptide and thus reduce or eliminate PBP5 localization to septal rings, resulting in an even distribution of PBP5 throughout the cell envelope. Replacing the active site serine with glycine at position 44 of PBP5 abolishes all *in vitro* penicillin binding as well as its D,D-carboxypeptidase activity (van der Linden *et al*., 1994). We constructed the Ser44Gly (S44G) mutant of PBP5 and expressed the cloned gene from a low copy number plasmid (pLP514). The D,D-carboxypeptidase-deficient strain CS703-1 and the wild-type LMC500 were transformed with pLP514 or with a plasmid expressing wild-type PBP5 (pLP515). These were grown in TY medium at 28°C and production of PBP5(S44G) was induced by addition of 0, 20 or 100 mM IPTG, after which the cells were grown for two MDs, harvested and fixed for immunolabelling. As determined by quantitative immunoblotting, the amount of PBP5 protein produced by these cells was increased by two to ten-fold compared with PBP5 in uninduced cells (not shown). At these levels, wild-type PBP5 complemented the morphological aberrations normally visible in the D,D-carboxypeptidase-deficient strain CS703-1, whereas the PBP5(S44G) protein did not complement this phenotype (not shown). Immunolabelling showed that PBP5(S44G) was redistributed from its midcell position to the lateral wall when compared with the localization pattern of wild-type PBP5 (Fig. 6A), suggesting that PBP5 localization at midcell depended on its ability to interact with its substrate.

We sought to confirm this result in live cells by observing the localization of the active site mutant when fused to sfGFP. DsbA(SS)-sfGFP-PBP5(S44G) was expressed in wild-type *E. coli*, in the Δ*dacA* strain CS12-7, and in the D,D-carboxypeptidase-deficient strain CS703-1. In all cases, sfGFP-PBP5(S44G) was evenly distributed in the envelope with either no or very weak fluorescence at midcell (Fig. 6B and D). Aztreonam-induced cell filaments of these strains did not show any midcell localization of mutant PBP5 despite the presence at midcell of a mature but inactive divisome (Fig. 6C and E). In fact, in many cells there was a visible dearth of fluorescently labelled sfGFP-PBP5(S44G) protein at sites where divisomes would be expected to be present (Fig. 6C and E), as if the mutant protein was avoiding these sites. The reason for this behaviour is not clear, but might be due to the inability of this protein to compete for binding to its substrate.

The S44G mutation is located deep in the cleft of the PBP5 active site, so it is highly unlikely that this change alters any putative protein–protein interaction between PBP5 and one of the divisome proteins. Therefore, we conclude that PBP5 localization to nascent septa depends in large measure on its ability to covalently bind and hydrolyze its pentapeptide substrate present in newly synthesized peptidoglycan.

### PBP5 localizes predominantly at the division site in spherical cells

If the localization of PBP5 to the septal ring was being driven by the location of its pentapeptide substrate, then the distribution of PBP5 throughout the rest of the periplasmic space might also be determined by active peptidoglycan synthesis. To test this idea, we inhibited lateral cell wall synthesis so that new pentapeptide substrates would appear only at developing septa. If substrate availability was important for PBP5 localization, then under such conditions the majority of PBP5 should relocate to these division sites.

**Fig. 7:**
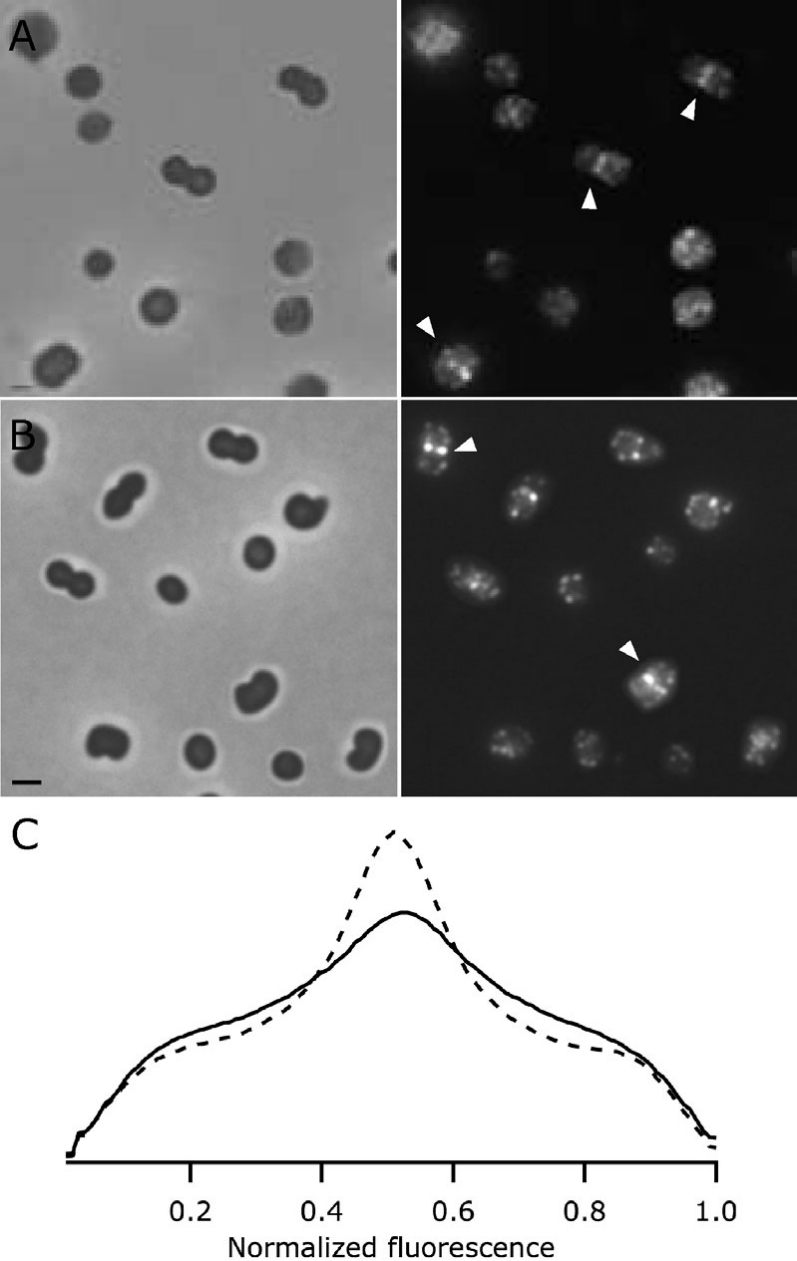
PBP5 localizes predominantly at the division site in spherical cells. A. Immunolocalization pattern of PBP5 in the wild-type strain LMC500 after inhibiting PBP2 by growing cells in the presence of 2 µg ml^−1^ mecillinam for two MDs. B. Immunolocalization pattern of PBP5 in the PBP2(Ts) strain LMC582 after inactivation of PBP2 by growing at the restrictive temperature for two MDs. Phase contrast images are on the left and fluorescence images are on the right. The bar equals 1 µm. C. Average fluorescence profiles per cell plotted against their normalized cell length. The profile of the dashed line is from the culture of spherical cells as shown in ‘A’ after growth for two MDs in the presence of mecillinam (*n* = 212). The profile of the solid line is from spherical cells as shown in ‘B’ after inactivating temperature-sensitive PBP2 by growing at 42°C for two MDs (*n* = 195).

We inhibited bacterial elongation by interfering with the function of PBP2, either by inactivating a temperature-sensitive mutant or by growing cells in the presence of mecillinam, a specific inhibitor of PBP2 (Park and Burman, 1973). Both treatments inhibit cell elongation so that *E. coli* grows as spherical cells. *E. coli* LMC582 carries the chromosomal mutation *pbpa137*(ts), which encodes a temperature-sensitive variant of PBP2 (Woldringh *et al*., 1988). This strain was grown in GB1 medium at 28°C until the cells were in log phase and then the culture was shifted to 42°C for two MDs, after which the cells were fixed, permeabilized and labelled with affinity-purified IgG antibody against PBP5. The amount of PBP5 was reduced around the circumference of each cell but showed a clear increase in intensity at midcell sites of ongoing cell division (Fig. 7B and C solid line). To determine whether the increased midcell localization of PBP5 was due to the absence of PBP2 (after thermal inactivation) or due to the lack of PBP2 enzymatic activity, we monitored the distribution of PBP5 by immunolocalization in the parent strain LMC500 after growth in the presence of mecillinam, which inhibits PBP2 activity without destroying the protein. LMC500 cells were grown to steady state in GB1 medium, mecillinam (2 µg ml^−1^) was added, and growth was allowed to continue for two MDs. The morphology and PBP5 localization pattern in cells treated with mecillinam were almost indistinguishable from the morphology and pattern observed in the thermosensitive PBP2 strain LMC582 (Fig. 7A and C dashed line). In these cells, the majority of envelope growth is derived from the synthesis of the two new cell poles during septation. Therefore, the similarity of PBP5 localization in the PBP2(ts) strain and in mecillinam-treated cells suggests that the reduction of PBP5 in the lateral wall does not reflect the absence of intact PBP2 but the absence of active peptidoglycan synthesis at those sites. It appears, then, that PBP5 is involved in cell wall elongation and septation and that the protein localizes at sites of active and ongoing peptidoglycan synthesis.

### PBP5 localization and function requires its membrane-anchoring domain

If the presence of pentapeptide substrates was the only determining factor for the localization of PBP5, then one would expect that a soluble periplasmic form of PBP5, which is unable to anchor itself to the inner membrane, would give the same localization pattern as that produced by the membrane-bound form of PBP5. To determine if this was the case, we constructed a PBP5 mutant that lacked the C-terminal 18 amino acids, which comprise the membrane-binding amphipathic helix (Pratt *et al*., 1986), and fused this construct to DsbA(SS)-sfGFP. The localization of the fusion protein, DsbA(SS)-sfGFP-PBP5CΔ18, was visualized in the parental strain CS109, in the PBP5 deletion strain CS12-7 and in the D,D-carboxypeptidases-deficient strain CS703-1 (Fig. 8). In the parent strain the protein localized evenly along the cell periphery and showed no preference for localization at the septum in constricting cells (Fig. 8A). Similar results were observed when the wild-type cells were treated with aztreonam (Fig. 8B). However, in 3% of cells (*n* = 195) lacking PBP5 (CS12-7), the DsbA(SS)-sfGFP-PBP5CΔ18 fusion protein localized weakly to the septum (data not shown). Because these two strains contain functional PBP5 and PBP6 (in CS109) or PBP6 (in CS12-7), it appears that removing the membrane anchor from PBP5 creates a protein that cannot compete efficiently with these membrane-bound D,D-carboxypeptidases for pentapeptide substrate at division sites. This interpretation was reinforced by the localization of DsbA(SS)-sfGFP-PBP5CΔ18 in *E. coli* CS703-1, which contains neither of these competing enzymes. In this strain, 26% of the cells (*n* = 240), showed a faint fluorescent band at the septum (Fig. 8D) indicating that at least some of the soluble protein was able to bind its substrate at midcell in the absence of competing proteins. Thus, we conclude that the amphipathic membrane anchor is required for optimal localization of PBP5.

We next asked if changes in the cellular localization of PBP5 were correlated with its physiological function. For this purpose we determined whether the ability of PBP5 to complement the morphological abnormalities of *E. coli* CS703-1 was altered by the presence or absence of the protein's membrane anchor. We measured the extent of cell shape deformities by using a Fluorescence Activated Cell Sorter, which can quantify the relative size and complexity (shape) of bacterial cells. We have previously used this procedure to identify even minor cell shape differences among *E. coli* strains lacking different combinations of PBPs (Meberg *et al*., 2004). When expressed in the morphologically complex mutant CS703-1, the membrane-anchored DsbA(SS)-sfGFP-PBP5 protein returned this strain to almost completely normal cell shape (Fig. S4B). In contrast, a fusion protein containing the active site mutant PBP5(S44G) did not complement the phenotype, and in fact seemed to make the cells more aberrant (Fig. S4C). The anchorless DsbA(SS)-sfGFP-PBP5CΔ18 protein produced only a slight complementation of the aberrant morphology in this strain (Fig. S4D, compared with Fig. S4A). These observations agree with previous work employing native PBP5 constructs that were not fused to GFP (Nelson *et al*., 2002). As stated previously, when expressed in *E. coli* CS703-1 cells grown for one MD in the presence of aztreonam, the anchorless PBP5CΔ18 fusion protein localized to midcell in 26% of the population. However, the anchorless form of the active site mutant, PBP5(S44G), localized evenly around the cell periphery but in many cases avoided the midcell position (Fig. S5), where wild-type PBP5 (or PBP5CΔ18) would normally accumulate. This avoidance mechanism is peculiar and is under investigation, but may mean that the active site mutant has a greatly reduced affinity for the kinds of substrates present at the septum. In any case, the results show that efficient septal localization of PBP5 requires both the amphipathic membrane anchor plus an intact active site.

We conclude that differences in the *in vivo* function of PBP5 are not determined solely by its enzymatic activity but are instead correlated with differences in cellular localization. Biologically functional PBP5 localizes to sites of active peptidoglycan synthesis whereas non-functional forms of PBP5 are spread diffusely in the periplasm. In particular, PBP5 must be anchored to the outer face of the inner membrane for the protein to localize properly and be biologically functional.

### PBP5 activity is not very substrate-specific

Because substrate recognition seemed to be essential for PBP5 localization, differences in substrate specificity might help explain how this enzyme assists in the regulation of cell elongation and division. To determine whether the difference in physiological function of the soluble and membrane-bound forms of PBP5 could be explained by changes in their enzymatic activities, we examined the substrate specificity of these proteins towards a number of artificial and natural substrates.

**Fig. 9:**
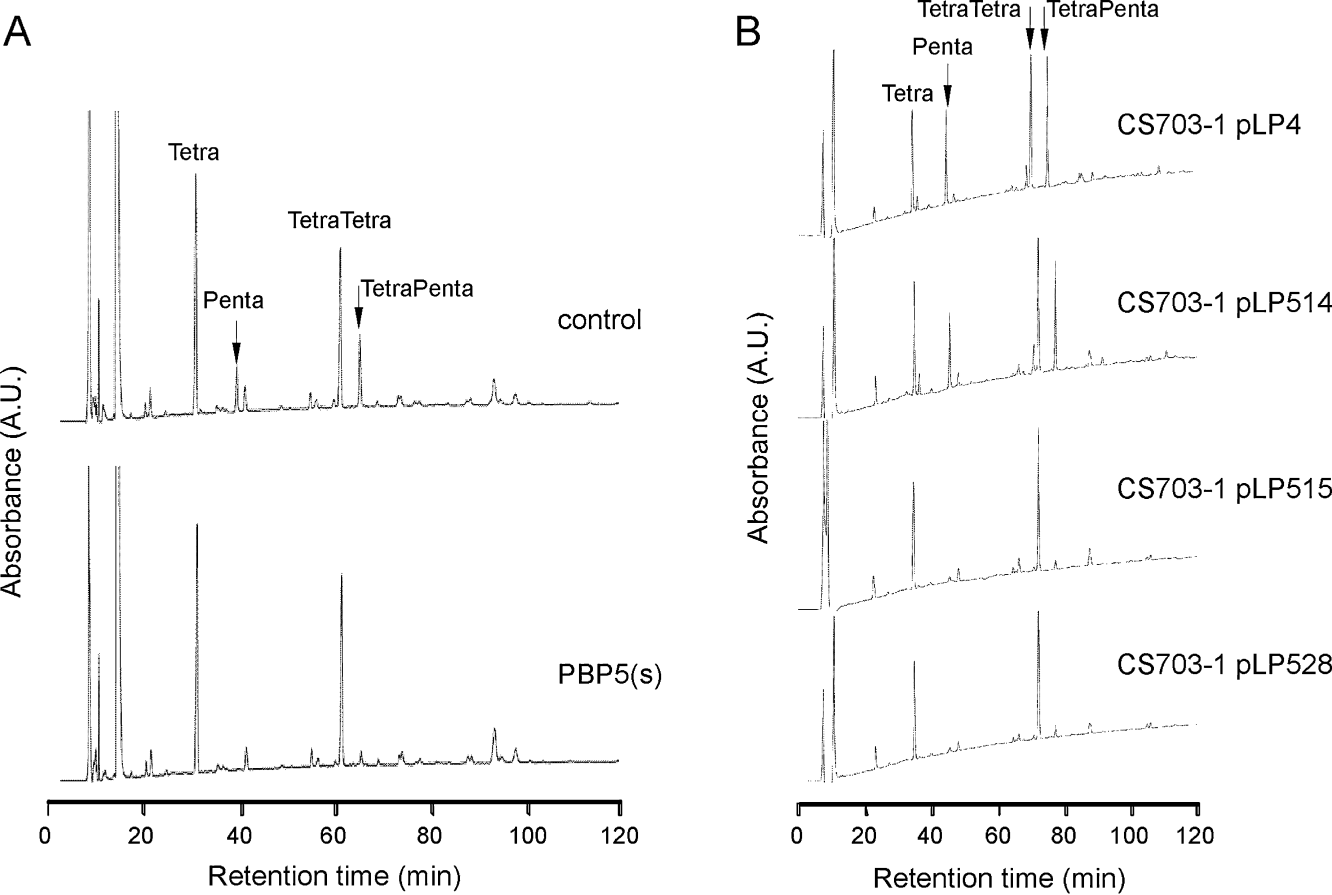
Activity of different forms of PBP5 towards whole cell sacculi. A. *In vitro* enzymatic activity of soluble PBP5(s) against pentapeptides present in isolated peptidoglycan sacculi. Peptidoglycan sacculi from strain D456 were incubated with PBP5(s) and digested with cellosyl followed by HPLC analysis of the muropeptide profile. Control is a sample without PBP5(s). Tetra, disaccharide tetrapeptide; penta, disaccharide pentapeptide; TetraTetra, bis-disaccharide tetratetrapeptide; TetraPenta, bis-disaccharide tetrapentapeptide. B. *In vivo* enzymatic activity of membrane-associated and soluble, periplasmic PBP5 in *E. coli* cells. Muropeptides from the pentapeptide-rich strain CS703-1 expressing membrane-associated PBP5 (from plasmid pLP515), soluble, periplasmic PBP5CΔ18 (from pLP528) or an inactive, membrane-associated version, PBP5(S44G) (from pLP514), were analysed by HPLC. pLP4 is the empty control plasmid. Membrane-associated and soluble PBP5 were active against pentapeptides present in sacculi in growing cells.

The water-soluble form of PBP5 exhibited an active D,D-carboxypeptidase activity with a clear preference for UDP-MurNAc-pentapeptide, although substrate S2d (benzoyl-D-Ala-thiolglycolate, the lateral chain of penicillin) was also hydrolyzed (Table 1 and Table S2). PBP5 reacted with several of these substrates with a kcat (Table 1) close to the expected kcat of 4 s^−1^ (Pratt, 2008). Soluble PBP5 also hydrolyzed lipid II and isolated sacculi (Fig. S6 and Fig. 9). These results suggest that the soluble form of PBP5 does not require the disaccharide moiety of the peptidoglycan-building unit for recognizing its pentapeptide substrate (see for a review on substrate specificity of D,D-peptidases: Pratt, 2008).

**Table 1:** Specificity profile and kinetic parameters of the hydrolysis reaction catalysed by purified water soluble PBP5.

Substrate^a^	k_cat_ (s^−1^)	K_m_ (mM)	k_cat_/K_m_ (M^−1^ s^−1^)
Ac_2_KAA	2.16	17	130
α AcKAA	0.79	9.5	1.39
UDPMurNAc-pentapeptide	1.93	0.05	27800
S1e	0.17	3.7	40
S2a	3.9	0.6	4800
S2d	24.6	0.74	33000
S2c	1.0	0.3	2650
S2val			12
S2leu			22
S2Phe			—

a Ac_2_KAA is bis-acetyl-L-Lys-D-Ala-D-Ala, α AcKAA is α-acetyl-L-Lys-D-Ala-D-Ala, UDPMurNAc-pentapeptide is L-Ala-γ-D-Glu-mesoA2pm-D-Ala-D-Ala. S stands for CO-C_6_H_5_-NH-CHR1-CO-X-CHR2-COO^−1^ with S1e R1 is H, R2 is C_6_H_5_-CH_2_ and X is O. For all other substrates X is S. For substrate S2a R1 and R2 are an H, substrate S2c R1 is H and R2 is CH_3_(D), substrate S2d R1 is CH_3_(D), and R2 is H. In case of Val, Leu, or Phe R1 is a mixture of the L and D amino acid and R2 is H [see also (Wilkin *et al*., 1994)]. Standard deviation values are 15% or less.

To determine if binding to membrane altered the substrates on which PBP5 could act, we removed the signal sequence from PBP5 and replaced it with an N-terminal oligo-histidine tag. This His-PBP5 construct remained in the cytoplasm but retained its C-terminal amphipathic helix, and the protein was recovered, as expected, in the membrane fraction of broken cells (not shown). His-PBP5 was purified by metal affinity chromatography, followed by removal of the His-tag by cleaving with thrombin and purification by cation exchange chromatography. The purified protein (Fig. S7) was incubated with different possible natural substrates containing D-Ala-D-Ala residues. This form of PBP5 removed terminal D-Ala residues from pentapeptides present in peptidoglycan sacculi of strain D456 (Fig. S7C), from the peptidoglycan precursor lipid II (Fig. S7B) and from the cytoplasmic precursor UDP-MurNAc pentapeptide (not shown). Also, solubilized pentapeptide-containing muropeptides from strain D456 were digested quantitatively (not shown). Monomeric and dimeric (cross-linked) pentapeptides in sacculi as well as individual monomeric and dimeric muropeptides served as PBP5 substrates. As expected, the enzymatic activity of this full-length PBP5 was completely abolished by pre-incubating the protein with ampicillin (not shown). Thus, like its soluble counterpart, PBP5 containing its carboxy-terminal amphiphatic helix was active against a spectrum of different substrates, including high molecular mass peptidoglycan, its soluble degradation products and peptidoglycan precursors (Table 2). We conclude that the soluble and membrane-bound forms of PBP5 recognize the same range of substrates and that PBP5 does not discriminate between muropeptides derived from different sources (i.e. from the sacculus, lipid-II, UDPMurNAc-pentapeptide or ampicillin).

**Table 2:** Specificity profile of the hydrolysis reaction catalysed by full-length PBP5.

	% decrease in	
Substrate	Penta monomer	TetraPenta dimer
Muropeptides^a^	97	83
Isolated murein^a^	100	46
Murein^b^ synthesized by PBP1B	59	38

a Muropeptides and isolated sacculi were from strain D456 that is enriched in pentapeptides. Buffer was 50 mM sodium Phosphate pH 6.0 and 0.05% T X-100.

b Newly synthesized murein was made by PBP1B with lipid II as substrate.

Although the soluble (non-membrane-bound) form of PBP5 (PBP5CΔ18) exhibited full enzymatic activity, it did not complement the morphological aberrations in *E. coli* strains lacking endogenous PBP5 (see above). It was possible that if PBP5 were not anchored to the membrane then the protein might not be able to perform its proper D,D-carboxypeptidase activity *in vivo*. For example, the peptidoglycan layer is stretched by osmotic pressure in living cells whereas isolated sacculi are fully relaxed, and this might make the substrate inaccessible to PBP5CΔ18. Alternately, PBP5CΔ18 might not be able to reach pentapeptide attached to nascent glycan strands in live cells if these substrates were concealed by peptidoglycan synthetases in the divisome. To address these questions, we compared the amounts of pentapeptide remaining in sacculi of the D,D-carboxypeptidase-deficient strain *E. coli* CS703-1 in which we expressed no PBP5 (vector control), wild-type PBP5 (from pLP515), inactive PBP5 (PBP5S44G from pLP514), or soluble, periplasmic, non-membrane-bound PBP5 (PBP5CΔ18 from pLP528) (Fig. 9B). Soluble, periplasmic, non-membrane-bound PBP5 digested pentapeptides just as efficiently as wild-type PBP5, while inactive PBP5S44G exhibited no activity. We conclude that the inability of PBP5CΔ18 to complement the morphological defects of *E. coli* CS703-1 cannot be explained by the inability to access its substrates *in vivo*. Therefore, because the anchorless protein is otherwise fully active, the proper localization and physiological function of PBP5 must depend on its ability to bind the outer face of the inner membrane as well as on its enzymatic activity.

## Discussion

### Cellular localization of PBP5

We report that PBP5 localizes to the cylindrical cell wall of *E. coli*, to sites of ongoing cell division and to nascent division sites before constriction is visible. Furthermore, the mechanism by which PBP5 is localized, especially at developing septa, depends on the presence of pentapeptide substrates in the wall, on a functional active site in the enzyme, and on the attachment of periplasmic PBP5 to the outer face of the inner membrane. Finally, the data strongly suggest that the normal cellular morphology of *E. coli* requires not only that PBP5 be active but also that it is properly localized to the division site.

Several strands of evidence support the contention that PBP5 localization is driven by the presence of its pentapeptide substrate. New peptidoglycan-building units, the GlcNAc-MurNAc-pentapeptides, are inserted in dispersed fashion into the old peptidoglycan layer in the cylindrical part of the cell during elongation, whereas new cell poles are composed only of newly inserted material (de Pedro *et al*., 1997). This means that the concentration of pentapeptide precursors is much higher at the division site than that in the cylindrical part of the cell wall. The localization of PBP5 mirrored this distribution, in particular its high concentration at newly forming septa. Of course, such a pattern might be driven by protein–protein interactions, but several additional observations argue against this possibility. First of all, the active site mutant PBP5(S44G) did not localize specifically to midcell positions even in the presence of pentapeptide substrates. Because the active site is embedded within the globular domain of PBP5, it is highly unlikely that the mutation altered any putative protein–protein interactions. Therefore, the inability to localize to the septum is best explained by the inability to covalently bind and hydrolyze its substrate.

Second, in spherical cells, in which peptidoglycan synthesis in the cylindrical portion of the cell wall is inhibited, PBP5 moves from its normal position in the lateral wall and accumulates at division planes, which are the only sites of ongoing peptidoglycan synthesis. This strongly suggests that PBP5 localization is determined to a large extent by where its substrate is being inserted. It is noteworthy that this redistribution of PBP5 occurs either when a temperature-sensitive PBP2 protein is inactivated or when the enzymatic activity of wild-type PBP2 is inhibited, thus reducing the likelihood that PBP5 interacts with PBP2 or with any other protein engaged in lateral cell wall elongation.

A third line of evidence is that in cells in which different stages of cell division are inhibited, PBP5 accumulates at sites of pentapeptide synthesis and insertion. Two types of septal peptidoglycan synthesis can be discriminated: preseptal peptidoglycan synthesis that requires the presence of an FtsZ ring but not a mature divisome (Nanninga, 1991; de Pedro *et al*., 1997; Ishidate *et al*., 1998; Varma and Young, 2004; 2009; Varma *et al*., 2007), and the subsequent synthesis of new cell poles by the combined activities of PBP3 and PBP1B (Bertsche *et al*., 2006). In the first type, peptidoglycan synthesis is initiated by the polymerization of FtsZ at midcell well before a septal constriction is visible (Den Blaauwen *et al*., 1999). We find that PBP5 localization at midcell depends on the presence and activity of FtsZ but not on the presence of FtsA at this stage. The second type of peptidoglycan synthesis is inhibited by aztreonam, which acts on the divisome protein PBP3, or by inactivation of temperature-sensitive mutants of PBP3. Inhibiting PBP3 reduces the amount of peptidoglycan insertion at midcell, and in such cells a thin band of PBP5 still accumulates at stalled division sites, consistent with the deficiency of pentapeptides in these areas. The localization of PBP5 at midcell in these filaments must be driven by the appearance of pentapeptides created by FtsZ-supported glycan strand synthesis. Furthermore, in filaments created by growing cells in the presence of aztreonam for two MDs, division at midcell is aborted but FtsZ initiates two new Z-rings at the one-quarter and three-quarter length positions of these cells, and neither of the new FtsZ rings contain any accompanying PBP3 (Den Blaauwen *et al*., 2003; Bertsche *et al*., 2006). However, bands of PBP5 still relocate to these future division sites, supporting the notion that PBP5 localizes because of FtsZ-supported glycan strand synthesis. Overall, the results indicate that protein–protein interactions with components of the mature divisome are not required for PBP5 localization, and provide substantial evidence that PBP5 localization is driven by the presence of its substrate.

### Substrate-driven localization of PBPs from other organisms

The dependence of PBP5 localization on the presence of its substrate is paralleled by the behaviour of a few PBPs in other organisms. The Class A PBP2 of *Staphylococcus aureus* localizes at the septum of this spherical gram positive bacterium (Pinho and Errington, 2003), but inhibiting the transpeptidase activity of PBP2 by the addition of oxacillin dislodges PBP2 from the septum and spreads it over the entire cell wall (Pinho and Errington, 2005). New peptidoglycan is still produced under these conditions, proving that the lack of midcell localization for PBP2 is not due to the absence of its substrate but rather due to the inability of PBP2 to bind substrate in the presence of oxacillin. PBP2 localization also becomes dispersed when the production of septal pentapeptide precursors is inhibited by the addition of cycloserine, further supporting the idea that PBP2 localization is determined by the presence of its substrate (Pinho and Errington, 2005). Similarly, in the American football-shaped bacterium, *Streptococcus pneumonia*, the Class A and B PBPs are delocalized from the central FtsZ-ring in a mutant deficient in PBP3, the D,D-carboxypeptidase of this organism (Morlot *et al*., 2004). PBP3 is normally located everywhere in the cell wall except at the central division plane, meaning that pentapeptide substrates will accumulate only at that site. In the PBP3-deficient mutant, then, pentapeptide substrates are available at many positions other than just at midcell, and this apparently disrupts the specific localization of high molecular mass PBPs, and cell separation is severely affected in this organism (Morlot *et al*., 2004). Therefore, analogous to what we now show for *E. coli* PBP5, substrate availability directs the cellular localization of PBP2 in *S. aureus* and of the Class A and B PBPs in *Streptococcus pneumoniae*.

Although the localization of other PBPs has been investigated in *Bacillus subtilis* (Scheffers and Pinho, 2005) and in *E. coli* (Weiss *et al*., 1999; Den Blaauwen *et al*., 2003; Bertsche *et al*., 2006), only in case of *E. coli* PBP3 and PBP1B, has it been established that the proteins do not require substrate for septal localization (Weiss *et al*., 1999; Bertsche *et al*., 2006). Thus, it is possible that the localization of other PBPs might also be codependent on substrate availability. This may help explain the relative ease with which various redundant PBPs can functionally replace one another.

### Membrane attachment is required for midcell localization and biological function of PBP5

Although wild-type PBP5 localizes strongly to septal sites, the soluble form PBP5CΔ18 (lacking the C-terminal membrane anchor), is evenly distributed and does not localize to division planes in cell filaments in which PBP3 is inhibited. This means that the ability to bind substrate does not by itself ensure that PBP5 will localize properly. Instead, PBP5 must be correctly tethered by its amphipathic helix to the outer surface of the inner membrane if the protein is to make productive contact with its substrate. On the other hand, the PBP5CΔ18 protein does localize, although weakly, to midcell positions in cells of the D,D-carboxypeptidase-deficient strain, CS703-1. This suggests that the untethered form of PBP5 can still find and bind its substrate if competing proteins are absent. In short, PBP5 lacking its membrane anchor seems unable to compete efficiently with other D,D-carboxypeptidases for substrate, and even if these competitors are absent it is apparently difficult for PBP5 to access nascent glycan strands without being properly attached to the inner membrane.

Not only does a PBP5 mutant lacking its membrane-binding C-terminus not localize properly, it also does not complement the morphological aberrations of an *E. coli* strain that lacks D,D-carboxypeptidase activity (Nelson *et al*., 2002). The simplest interpretation is that membrane localization of PBP5 is required for its morphological function. In fact, complementation can be restored to the anchorless PBP5 mutant by fusing it to the analogous C-terminus of either PBP6 or DacD, but complementation is not restored by adding the membrane anchor of PBP4 or an unrelated membrane spanning helix (Nelson *et al*., 2002). Therefore, the biological activity of PBP5 is not determined simply by being attached to the membrane. Instead, PBP5 must be attached in a particular way. For example, the PBP5 membrane anchor differs structurally from that of PBP4. The PBP5 C-terminal anchor forms an obliquely oriented α-helix (O'Daniel *et al*., 2010) that penetrates deeper into the membrane compared with the amphipathic helix of PBP4 (Harris *et al*., 2002; O'Daniel *et al*., 2010), which could explain the functional difference. Because substrate localization seems to drive PBP5 localization, we speculate that the PBP5 membrane anchor positions the enzyme so that its substrate becomes accessible.

As an alternative interpretation, it could be argued that removing the membrane anchor from PBP5 alters its D,D-carboxypeptidase activity so that it no longer binds efficiently to the pentapeptide substrate. If so, this might explain why PBP5 neither localizes properly nor fulfils its morphological function. Our results argue against this alternative. Soluble (anchorless) PBP5 and membrane-bound PBP5 are equally capable of removing D-alanine residues from a range of substrates, including the lipid-bound peptidoglycan precursor lipid II, molecules that mimic the peptide side chain, and isolated sacculi and sacculus in the living cell. These observations make it unlikely that soluble PBP5 has a reduced affinity for muropeptides. In addition, the lack of substrate specificity indicates that the active site of PBP5 does not discriminate between various types of cross-linked murein (Stefanova *et al*., 2002), the peptidoglycan layer or nascent glycan strands. Even so, soluble PBP5 is only marginally able to complement the D,D-carboxypeptidase-deficient mutant strain CS703-1, indicating that the problem does not lie in an enzymatic defect of PBP5. Instead, although it retains a normal range of enzymatic activity, anchorless PBP5 is unable to functionally interact with its proper pentapeptide substrate because it is not positioned appropriately on the inner membrane.

### Function of PBP5

**Fig. 10:**
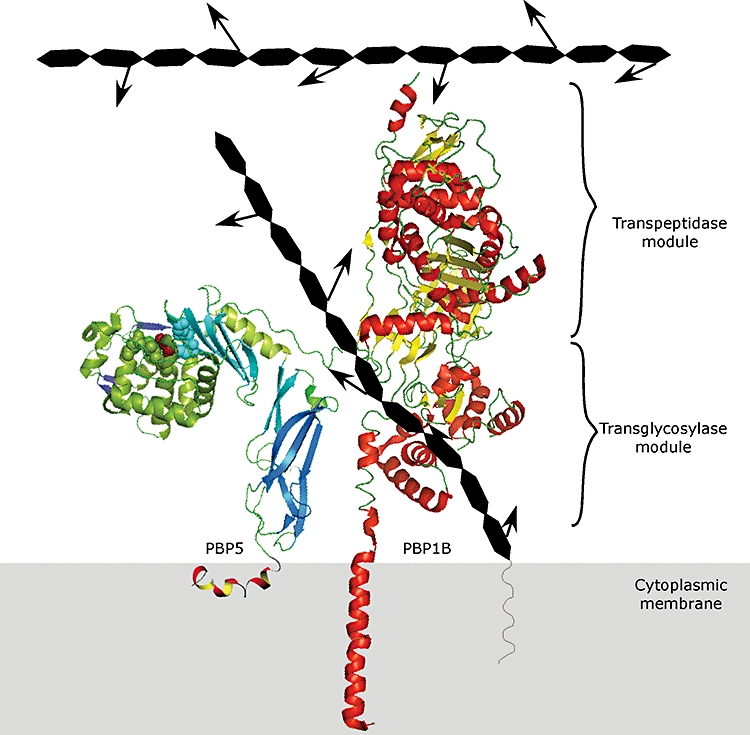
Model showing possible spatial organization of PBP5 and PBP1B with respect to peptidoglycan substrates and products. The crystal structures of the two PBPs are drawn to scale to one another. The depth of the cytoplasmic membrane (lower grey rectangle) and the location of the glycan chains (hexagons) are not drawn to scale but merely illustrate their relative locations with regard to the PBPs. Because it does not reach as high into the periplasm, membrane attached PBP5 could not interact with the existing peptidoglycan layer (upper hexagonal chain) but instead have access only to pentapeptide substrates (arrows) on the newly synthesized glycan chain (diagonal hexagonal chain).

*In vitro*, PBP5 has D,D-carboxypeptidase activity in the absence of other proteins, indicating that interactions with proteins present in the elongasome or divisome are not required for its activity. Instead, ongoing glycan strand synthesis by Class A and B PBPs during cell elongation or division seems to be all that is necessary to provide the substrate on which PBP5 acts. The recently published crystal structure of PBP1B (Sung *et al*., 2009) shows that the transglycosylase reaction occurs relatively close to the cytoplasmic membrane, whereas the transpeptidation takes place in the upper most part of the protein. The active site of PBP5 is approximately located at a distance of 60 Å from the surface of the cytoplasmic membrane (Nicholas *et al*., 2003). During the growth of the nascent glycan strand before the strand is inserted into the existing peptidoglycan layer, the pentapeptide substrate will potentially be available to PBP5 before it reaches the pentapeptide-binding site of PBP1B about 100 Å from the membrane surface (Fig. 10). If the hinge region between the pedestal module II and the enzymatic active domain I of PBP5 allows free rotation, the protein could even have access to pentapeptides at different angles of the helically synthesized glycan strands. A similar line of thought can be applied to PBP3 in which the transpeptidase domain is at comparable height to that of PBP1B (Gordon *et al*., 2000). Assuming that the Class A and B PBPs are not protruding through the sacculus but that the peptidoglycan is stretched above these proteins, PBP5 would not be able to reach pentapeptides in the existing peptidoglycan layer and its activity would be limited to the nascent glycan strands synthesized by the Class A PBP. This would also explain why PBP5 needs to be membrane-bound to perform its function. Removal of D-alanines from the existing peptidoglycan layer as performed by the soluble PBP5 does not, as we show, assist in maintaining the correct bacterial shape.

Being properly positioned, then, enables PBP5 to remove terminal D-alanine residues from the correct positions of newly synthesized glycan strands, which in turn regulates of the number and kinds of possible peptide cross links in the peptidoglycan layer. In contrast, soluble PBP5 would remove whatever D-alanine it encounters at random positions on the nascent glycan strands and in peptidoglycan. This may put the soluble enzyme in direct competition with the Class A and B PBPs for pentapeptides required for peptide cross-linking. In fact, overproducing PBP5 produces spherical growth of *E. coli* after a prolonged period of time (Markiewicz *et al*., 1982), whereas overproducing the soluble form of PBP5 produces spherical cells within a few generations (L. Potluri and K.D. Young, unpublished). Thus, the soluble form of PBP5 is a more potent inhibitor of cell wall elongation, consistent with what would be expected if this protein had access to a greater number of substrates.

In summary, we conclude that the cellular localization of PBP5 is determined predominantly by sites of active peptidoglycan synthesis, which provide pentapeptide substrate that are accessed most efficiently by the membrane-bound form of PBP5. In turn, this highly specific localization of D,D-carboxypeptidase activity to the developing septum is required for proper maintenance of cell morphology. Just how and why PBP5 must be localized so specifically remain questions to be addressed further.

## Experimental procedures

### Bacterial strains and growth conditions

All *E. coli* strains used in this work are listed in Table 3. *E. coli* LP9-1 was constructed by P1 transducing the *ftsZ84*(ts) mutation into strain CS109, using phage lysate prepared from *E. coli* WM1109 (Geissler *et al*., 2003). *E. coli* LP10-1 was constructed by P1 transducing the *ftsI23*(ts) mutation into CS109, using phage lysate prepared from *E. coli* MCI23 (Dai *et al*., 1993). Both mutations are linked to the nearby *leu*::Tn10 allele. Transductants were selected on Luria-Bertani (LB) + tetracycline (10 µg ml^−1^) plates at 30°C and screened for filamentation at 42°C. *E. coli* LP28-1 carries the *ftsI23*(ts) mutation in the CS703-1 background. Interestingly, LP28-1 grows slowly and filaments in LB at 30°C but grows normally on LB with 0.5% NaCl at 30°C.

**Table 3:** Bacterial strains and plasmids.

	Relevant characteristics	Reference/Source
*E. coli* Strain		
LMC500 (MC4100)	F-, *araD139, Δ(argF-lac)U169deoC1, flbB5301, ptsF25, rbsR, relA1, rpslL150, lysA1*	(Taschner *et al*., 1988)
LMC509	MC4100 *lysA, ftsZ84(ts)*	(Taschner *et al*., 1988)
LMC510	MC4100 *lysA, ftsI2158(ts)*	(Taschner *et al*., 1988)
LMC512	MC4100 *lysA, ftsA12(ts)*	(Taschner *et al*., 1988)
LMC531	MC4100 *lysA, ftsQ1(ts)*	(Taschner *et al*., 1988)
LMC582	MC4100 *lysA, pbpa 137 (ts)*	(Woldringh *et al*., 1988)
CS109	W1485*, F thi glnV (supE) rph-1 rpoS*	(Denome *et al*., 1999)
CS12-7	CS109 *dacA*	(Denome *et al*., 1999)
CS703-1	CS109 derivative that lacks PBPs 1A, 4, 5, 6, 7, AmpC and AmpH	(Denome *et al*., 1999, Meberg *et al*., 2001)
D456	Triple deletion of PBP4, 5 and 6	(Edwards and Donachie, 1993)
WM1109	MG1655 *ftsZ84(ts) leu::Tn10* (tetR)	(Geissler *et al*., 2003)
PS236	W3110 *ftsA12(ts) leu::Tn10* (tetR)	(Pichof and Lutkenhaus, 2002)
MCI23	MC4100 *ftsI23(ts) leu::Tn10* (tetR)	(Dai *et al*., 1993)
LP9-1	CS109 *ftsZ84(ts) leu::Tn10* (tetR)	This work
LP10-1	CS109 *ftsI23(ts) leu::Tn10* (tetR)	This work
LP28-1	CS703-1 *ftsI23(ts) leu::Tn10* (tetR)	This work
Plasmid		
pDACAhis	pET28a(+) derivative that encodes MGSSH_6_SSGLVPRGSHMAS-PBP5(N33-G403)	This work
pMLB1113	Medium copy vector, P_lac_*lacI*^q^, Amp^R^	(de Boer *et al*., 1989)
pTB263	Plasmid containing the *dsbA*(SS)-*sfgfp* DNA sequence	T. Bernhardt
pLP4	P_lac_*lacI*^q^ Kan^R^	This work
pLP8	pLP4 with mutated HindIII site in Kan^R^	This work
pLP9	P_lac_–*dsbA*(SS)-*sfgfp lacI*^q^ Kan^R^	This work
pPJ5S-SDM	P_ara_-*dacA*-S44G	This work
pLP514	P_lac_–*dacA-S44G lacI*^q^ Kan^R^	This work
pPJ5	P_ara_-*dacA*	(Nelson and Young, 2000)
pLP515	P_lac_–*dacA lacI*^q^ Kan^R^	This work
pLP521	P_lac_–*dsbA*(SS)-*sfgfp-dacA lacI*^q^ Kan^R^	This work
pLP522	P_lac_–*dsbA*(SS)-*sfgfp-dacA-* S44G *lacI*^q^ Kan^R^	This work
pLP523	P_lac_–*dsbA*(SS)-*sfgfp-dacA-CΔ18 lacI*^q^ Kan^R^	This work
pLP524	P_lac_–*dsbA*(SS)-*sfgfp-dacA-S44G-CΔ18 lacI*^q^ Kan^R^	This work
pLP528	P_lac_–*dacA-CΔ18 lacI*^q^ Kan^R^	This work

For immunolabelling of *E. coli* LMC strains, cells were grown to steady state in glucose minimal medium (GB1) containing 6.33 g of K_2_HPO_4_.3H_2_O, 2.95 g of KH_2_PO_4_, 1.05 g of (NH_4_)_2_SO_4_, 0.10 g of MgSO_4_.7H_2_O, 0.28 mg of FeSO_4_.7H_2_O, 7.1 mg of Ca(NO_3_)_2_.4H_2_O, 4 mg of thiamine, 4 g of glucose and 50 mg of required amino acids, per litre pH 7.0 at 28°C. Because of the use of temperature-sensitive strains, 28°C was used as standard growth temperature. The strains LMC500, LMC509, LMC510, LMC512 and LMC582 required lysine, for growth in minimal medium. LMC509 was grown in a low-salt glucose minimal medium (1/2 GB1), containing the same ingredients as GB1 but differing in the amount of salt, which is 3.16 g of K_2_HPO_4_.3H_2_O and 1.48 g of KH_2_PO_4_ per liter, pH 7.0. Cultures were considered to be in steady state of growth if the ratio between optical density and number of cells remained constant over time (Fishov *et al*., 1995). Absorbance was measured at 450 nm (GB1) or 600 nm (TY) with a 300-T-1 spectrophotometer (Gilford Instrument Laboratories). Cell number was measured with an electronic particle counter.

Strains CS109 and CS12-7 were grown at 28°C in rich TY medium containing 10 g bacto-tryptone, 5 g yeast extract, 5 g NaCl, 15 mmol NaOH per liter or in LB broth supplemented with 50 µg ml^−1^ Kanamycin in the case of sfGFP-PBP5 expression. Strain BL21(DE3) pDACAhis was grown in LB autoinduction medium that contained per liter 10 g tryptone, 5 g yeast extract, 0.5 g glucose, 2 g α-lactose, 1 mM MgSO_4_, 3.3 g (NH_4_)_2_SO_4_, 6.8 g KH_2_PO_4_ and 7.1 g Na_2_HPO_4_. Bacteria were filamented by the specific inhibition of PBP3 (Sykes and Bonner, 1985) by the addition of 1 µg ml^−1^ of aztreonam (ICN, Biomedicals Ohio) freshly dissolved in saturated Na_2_CO_3_. Spherical growth was achieved by inhibition of PBP2 by 2 µg ml^−1^ mecillinam (Park and Burman, 1973; Den Blaauwen *et al*., 2003). Both antibiotics were added at an optical density (OD_450_) of 0.025, and the cultures were further grown in the presence of the antibiotic for two MDs. Subsequently, cells were harvested and fixed for phase contrast imaging and immunofluorescence labelling as described below.

### Flow cytometry

CS703-1 cells carrying appropriate plasmids were grown overnight at 30°C in LB-Kan medium. Next morning, overnight cultures were diluted 1:150 in LB-Kan and incubated at 30°C for 2 h, after which IPTG was added to a final concentration of 50 µM and incubated further for 1.5 h. At this time all the cultures reached an OD_600_ of ∼0.4. Cells were harvested by pelleting 1 ml of culture at 8000 × g, washed once in PBS, and resuspended in 1 ml of PBS. These cell suspensions were diluted 1:10 in PBS and analysed with a FACSCalibur flow cytometer (BD Biosciences, San Jose, CA) (UAMS flow cytometry core facility) using the forward scatter and side scatter settings. CellQuest Pro software was used for the data acquisition and analysis. The sample size of each culture was 50 000 cells. CS109/pLP9 and CS703-1/pLP521 cells were used as a guide to draw gate 1 (R1), which represents normal rod-shaped cells. Gate 2 (R2), representing abnormal cells, was drawn by using the distribution of CS703-1/pLP9 cells as a guide, and by excluding the area covered by gate 1.

### Plasmid construction

*PfuUltra* II Fusion HS DNA polymerase (Stratagene) was used to amplify polymerase chain reaction (PCR) fragments for cloning, according to the manufacturer's instructions. Standard DNA manipulation techniques were used for cloning genes into plasmids, and the sequence of each cloned gene was confirmed by DNA sequencing (MWG Biotech and by the UAMS DNA Sequencing Facility).

Plasmid pDACAhis was created as follows. A truncated version of the *dacA* gene was amplified by PCR using primers 5′-CGTGTGAATTCTTTAACCAAACCAGTGATGG-3′ and 5′-CCGATGCTAGCAATATCAAAACTATGATCCC-3′ and template-DNA from strain MC1061. The PCR product was cleaved with NheI and EcoRI and ligated into plasmid pUC19, which had been digested with the same restriction enzymes. The ligation mixture was transformed into strain DH5α and a transformant containing the correct plasmid was selected. The plasmid was isolated, restricted with NheI and EcoRI, and the fragment containing the *dacA* gene was ligated into expression plasmid pET28a(+) (Novagen). The resulting plasmid pDACAhis was transformed into strain BL21(DE3), re-isolated, and the correct sequence of the *dacA* gene was confirmed. BL21(DE3) pDACAhis allowed overproduction of cytoplasmic PBP5 with a N-terminal oligohistidine-tag replacing the signal peptide.

Plasmid pLP4 (*P_lac_ lacI^q^ kan*) was created from the medium copy vector pMLB1113 (*P_lac_ lacI^q^ bla*) (de Boer *et al*., 1989) by replacing the β-*lactamase* (*bla*) gene in pMLB1113 with a kanamycin resistance gene (*kan*). The *kan* gene was amplified by PCR from pBAD18-Kan with the primers (upstream) 5′-GATGTTACATTGCACAAGAT-3′ and (downstream) 5′-CGCGCTGCTCATGACTCAGCAAAAGTTCGATT-3′, and the PCR product was digested with BspHI. (The BspHI site is underlined. Note that pBAD18-Kan carries one BspHI site upstream of the *kan* gene so only a single new site needed to be added.) pMLB1113 was digested with BspHI to remove the DNA fragment containing the *bla* gene, and into its place was ligated the *kan* gene fragment.

Plasmid pLP9 (*P_lac_-dsbA*(*SS*)*-sfgfp lacI^q^ kan*) was created as follows. First, the HindIII site in the *kan* gene of pLP4 was modified with the QuikChange Lightning Site-Directed Mutagenesis Kit (Stratagene), by using the primer 5′- GGAAAGAAATGCATAAACTTTTGCCATTCTCACC-3′ and its reverse complement. The resulting plasmid (pLP8) carried the *kan* gene with a silent mutation (underlined) at codon 184 (lysine), which eliminated an internal HindIII site so that a single HindIII sequence remained in the multiple cloning site. Next, the *dsbA(SS)-sfgfp* gene fusion from pTB263 (T. Bernhardt, personal communication) was cloned between the EcoRI and HindIII sites of pLP8 to give plasmid pLP9. This plasmid served as the backbone for creating the N-terminal s*fgfp* gene fusions described below. The s*fgfp* construct encodes the *dsbA* signal sequence (*SS*) at its 5′ end, which allows the translocation of sfGFP from cytoplasm to the periplasm (T.G. Bernhardt, personal communication).

Plasmid pLP514 (*P_lac_-dacA-S44G lacI^q^ kan*) was created by inserting a DNA fragment containing the *dacA-S44G* mutant gene into pLP4 between the BamHI and PstI sites, placing the mutant PBP 5 gene under control of the *lac* promoter. The *dacA-S44G* DNA fragment was obtained by digesting pPJ5S-SDM (*P_ara_-dacA-S44G*) with BamHI and PstI (L. Potluri and K.D. Young, unpublished).

Plasmid pLP515 (*P_lac_-dacA lacI^q^ kan*) was created by cloning the wild-type *dacA* gene from pPJ5 (*P_ara_-dacA*) (Nelson and Young, 2000) into the BamHI and PstI sites of pLP4.

Plasmid pLP521 (*P_lac_*-*dsbA*(*SS*)*-sfgfp-dacA lacI^q^ kan*) was created by cloning the wild-type *dacA* gene between the BamHI and HindIII sites of pLP9. The *dacA* gene was amplified from pLP515, using the primers (upstream) 5′-GCTGGCGGATCC**GATGACCTGAATATCAAAACT**-3′ and (downstream) 5′-GCATGCAAGCTTCTTT**TTAACCAAACCAGTGATGGAA**-3′ (the BamHI and HindIII sites are underlined; bolded sequences represent portions of the amplified gene). The resulting DNA fragment does not encode the original 29 amino acid signal sequence (SS) of *dacA*.

Plasmid pLP522 (*P_lac_*-*dsbA*(*SS*)*-sfgfp-dacA-S44G lacI^q^ kan*) was generated in the same way as pLP521 except that *dacA-S44G* was amplified from pLP514 and inserted into the BamHI and HindIII sites of pLP9.

Plasmid pLP523 (P*_lac_*-*dsbA*(*SS*)*-sfgfp-dacA*Δ*C18 lacI^q^ kanR*) was created by amplifying a DNA fragment lacking the final 54 nucleotides of the *dacA* gene (*dacA*Δ*C18*) from pLP515, by using the primers (upstream) 5′-GCTGGCGGATCC**GATGACCTGAATATCAAAACT**-3′ and (downstream) 5′-GCATGCAAGCTTCTTT*TTA***GTTACCTTCCGGGATTTC**-3′[the BamHI and HindIII sites are underlined; bolded sequences represent portions of the amplified gene; and a stop codon (italicized *TTA*) was inserted in front of the HindIII site]. The PCR fragment was digested and cloned into the BamHI and HindIII sites of pLP9. The resulting gene fusion does not encode the last 18 amino acids of PBP5, so that the expressed fusion protein lacks the carboxy-terminal amphipathic helix.

Plasmid pLP524 (P*_lac_*-*dsbA*(*SS*)*-sfgfp-dacA-S44G-*Δ*C18 lacI^q^ kanR*) was generated in the same way as pLP523 except that *dacA-S44G* was amplified from pLP514 and inserted into the BamHI and HindIII sites of pLP9.

Plasmid pLP528 (P*_lac_-dacA*Δ*C18 lacI^q^ kan*) was created by amplifying a DNA fragment lacking the final 54 nucleotides of the *dacA* gene (*dacA*Δ*C18*) from pPJ5 (Nelson and Young, 2000) by using the primers (upstream) 5′-ATGCCATAGCATTTTTATCC-3′ (this sequence corresponds to pBAD vector backbone) and (downstream) 5′-GCATGCAAGCTTCTTT*TTA***GTTACCTTCCGGGATTTC**-3′[the HindIII site is underlined; the bolded sequence represents the portion of the amplified gene; and a stop codon (italicized *TTA*) was inserted in front of the HindIII site]. The PCR product was digested with BamHI and HindIII (via sequential digestion) and cloned into pLP8. The resulting plasmid encodes wild-type PBP5 without the carboxy-terminal amphipathic helix.

### Expression and purification of PBP5 for activity assays

A water-soluble form of PBP5 lacking the carboxy-terminal amphiphatic helix was produced (1 mg) and purified (Table S1) as described previously (Ferreira *et al*., 1988; van der Linden *et al*., 1992).

The membrane-bound form of PBP5 was isolated from the cytoplasm as follows: three litres of LB autoinduction medium containing 50 µg ml^−1^ kanamycin were inoculated with 30 ml of an overnight culture of BL21(DE3) pDACAhis. The cells were grown for 18 h at 25°C and harvested by centrifugation at 4°C. The cell pellet was resuspended in 40 ml of 25 mM Tris/HCl, 10 mM MgCl_2_, 1 M NaCl, 20% glycerol, pH 7.5. A small amount of DNase was added and the cells were broken by sonication with a Branson Digital Sonifier operating at 70% amplitude for 5 min. The cell membranes were pelleted by ultracentrifugation at 80 000 × *g* for 60 min at 4°C. The pellet was extracted with 30 ml of 25 mM Tris/HCl, 10 mM MgCl_2_, 1 M NaCl, 20% glycerol, 2% Triton X-100, pH 7.5 for 16 h at 4°C. The supernatant obtained after another ultracentrifugation was supplemented with 20 mM imidazole and incubated with 5 ml Ni-NTA superflow beads for 16 h at 4°C. The beads were settled in a gravity column and washed with 100 ml of 25 mM Tris/HCl, 10 mM MgCl_2_, 1 M NaCl, 20% glycerol, 0.2%, Triton X-100, 20 mM imidazole, pH 7.5. Retained proteins were eluted with 25 ml of 25 mM Tris/HCl, 10 mM MgCl_2_, 1 M NaCl, 20% glycerol, 0.2%, Triton X-100, 400 mM imidazole, pH 7.5. The protein was then dialysed to remove the imidazole.

For removal of the oligohistidine tag, 20 U of thrombin was added and dialysis continued for 24 h against 25 mM Tris/HCl, 10 mM MgCl_2_, 1 M NaCl, 20% glycerol, pH 7.5. Quantitative removal of the oligohistidine tag was confirmed by SDS-PAGE analysis. The protein was dialysed against 10 mM sodium acetate, 10 mM MgCl_2_, 500 mM NaCl, 20% glycerol, pH 4.8, followed by 1:5 dilution with dialysis buffer lacking NaCl but containing 0.2% Triton X-100. The protein was applied to a 1 ml High Trap SP HP column (GE Health Care) at a flow rate of 0.5 ml min^-1^ using an ÄKTA prime FPLC machine. A 60 min gradient from buffer A (10 mM sodium acetate, 10 mM MgCl_2_, 100 mM NaCl, 0.2% Triton X-100, pH 4.8) to buffer B (10 mM sodium acetate, 10 mM MgCl_2_, 2 m NaCl, 0.2% Triton X-100, pH 4.8) was applied and 1.5 ml fractions was collected and analysed by SDS-PAGE. Fractions containing PBP5 were combined. The sample was dialysed against 25 mM HEPES, 10 ml MgCl_2_, 500 mM NaCl, 20% glycerol, pH 7.5 and stored at −20°C.

### Antibody production and purification

One mg of the purified water-soluble form of PBP5 lacking the carboxy-terminal amphiphatic helix was sent to the Centre d'Economie Rurale (Marloie, Belgium) to raise antibodies. Four subcutaneous injections of a rabbit were made at days 1, 14, 28 and 56, and antiserum was recovered 80 days after the first injection.

Purified PBP5 protein was separated by SDS-PAGE and transferred to a nitrocellulose membrane. The membrane was blocked with 5% skim milk for 4 h and incubated overnight with 100 µl antiserum at 4°C. Subsequently, the membrane was washed three times for 5 min with PBS and incubated for 10 min while vigorous shaking with 60 µl of 0.1 M glycine (pH 2.5). The pH of the solution was neutralized with the addition of 8 µl 1 M Tris (pH 9.5). For storage, 5% BSA was added to the eluted affinity-purified antibodies.

To eliminate cross-reactivity of the affinity-purified antiserum, especially with the PBP6 and PBP6B/DacD proteins, the antiserum was absorbed against a total protein content extracted from a mutant strain (CS12-7) that does not produce the PBP5 protein. In brief, *E. coli* CS12-7 was grown overnight in TY medium. The culture was collected by centrifugation and resuspended in 1/5 volume of 0.9% NaCl and transferred to ice for 5 min. Acetone (−20°C) was added to the chilled cell suspension and the precipitate was allowed to sediment for 30 min. Next, the precipitate was collected by centrifugation at 10 000 × *g*, for 10 min. The residue was subsequently suspended in fresh acetone (−20°C), mixed vigorously, and allowed to sediment for 10 min and collected by centrifugation 13 000 × *g* for 5 min. The precipitate was transferred to a clean filter paper, spread and air-dried at room temperature. The affinity-purified antiserum against PBP5 was incubated with the precipitate for 4 h at 4°C (1/10 v/v), and centrifuged at maximum speed for 5 min. The supernatant was used as a source of antibodies in the immunolabelling experiments.

### Fixation and permeabilization of cells

*Escherichia coli* cells were fixed in 2.8% formaldehyde and 0.04% glutaraldehyde in growth medium. After 15 min of incubation at room temperature the cells were collected at 8.000 × *g* for 5 min and washed twice in PBS. Fixed cells were permeabilized by the addition of 0.1% Triton X-100 in PBS and incubated for 45 min at room temperature. All following centrifugations were performed at 4.500 × *g*. The cells were washed three times in PBS and incubated in PBS containing 100 µg ml^−1^ lysozyme and 5 mM EDTA for 45 min at room temperature. Finally, the cells were washed three times in PBS.

### Immunolocalization experiments

*Escherichia coli* cells were labelled with purified antibodies against PBP5 as described by den Blaauwen *et al*. (Den Blaauwen *et al*., 2003). In brief, fixed and permeabilized cells were incubated at 37°C with 0.5% (w/v) blocking reagents (Boehringer) diluted in PBS. After 30 min of incubation, antibodies against PBP5 diluted in blocking buffer (1/15 v/v) were added to the cells and further incubated for 60 min at 37°C. The cells were washed three times with PBS containing 0.05% (v/v) Tween-20. Next, the cells were incubated for 30 min at 37°C with secondary antibody Cy3-conjugated Donkey Anti-Rabbit IgG (Jackson ImmunoResearch Laboratories, USA). The cells were washed again three times in Tween-20-PBS and resuspended in PBS.

### Microscopy and image analysis

For immunolocalization imaging the cells were immobilized on 1% agarose (Koppelman *et al*., 2004), and photographed with a CoolSnap *fx* (Photometrics) CCD camera mounted on an Olympus BX-60 fluorescence microscope through an UPLANFl 100x/1.3 oil objective (Japan). Images were taken using the program ‘Object-Image2.19 by Norbert Vischer (University of Amsterdam, http://simon.bio.uva.nl/Object-Image/object-image.html), which is based on National Institutes of Health Image-J by Wayne Rasband. In all experiments the cells were first photographed in the phase contrast mode, then with the Cy3 filter (U-MNG, ex. 530–550 nm). Next, the two photographs were stacked and the length and diameter of the cells were determined from the phase contrast images and the localization and intensity of the fluorescence signal was analysed in the fluorescence images of bacteria as described (Den Blaauwen *et al*., 2003; Bertsche *et al*., 2006). The fluorescence profiles of the cells were analysed using the public domain program ‘Object-J 0.98a’ by Norbert Vischer (University of Amsterdam, http://simon.bio.uva.nl/objectj/), which is based on image-J by Wayne Rasband. The map of profiles macro is used to create the average fluorescence profile of the indicated number of cells plotted against the normalized cell length. From each cell's axis marker, a band selection in the fluorescence channel with fixed line width of 20 pixels is created. For example, one cell with an axis length of 3 mm = 47 pixels, which created a profile array of 47 elements, each containing the mean value of 20 pixels perpendicular and symmetric to the axis. The array was resampled from 47 with interpolation to a length of 100 elements. Resampling can be done either with normalize Profile = true (keeping the peaks at same height as is done throughout the manuscript) or with normalize Profile = false (keeping the sum of all elements constant). The profiles for all cells are added and divided by the number of cells to obtain the average profile. All cells of which the fluorescence profiles are compared with each other were imaged using the same microscope setting. For optimal comparison of the shape of the profiles the surface below the profiles have been normalized to 1, unless stated otherwise.

For GFP localization, bacteria were grown overnight at 30°C in LB-Kan and the next day were diluted 1:250 (for CS703-1) or 1:500 (for CS109) into LB-Kan. After 2 h of growth at 30°C, isopropyl β-D-thiogalactoside (IPTG) was added to a final concentration of 50 µM to induce expression of the relevant sfGFP-tagged protein, and the cultures were incubated for another 110 min (CS109) or 135 min (CS703-1). When appropriate, aztreonam (1 µg ml^−1^) was added 1.15 h (CS109) or 1.5 h (CS703-1) after IPTG induction and the cultures were incubated for one MD. The temperature-sensitive strains LP9-1 and LP10-1 were grown in LB with 0.5% NaCl for 2 h, after which 20 µM IPTG was added and the cells were incubated at the permissive temperature (30°C) for one MD. The cells were shifted to the non-permissive temperature (42°C) and incubated further for one or two MDs. Cells from 0.5 ml of culture were concentrated by centrifugation at 2500 × *g* for 30–45 s, after which the pellet was resuspended in 100–200 µl of LB or LB-aztreonam. A portion of this suspension (5 µl) was spotted onto a microscope slide coated with 1% agarose and incubated at room temperature for 10 min to immobilize the cells. Slides were stored at 4°C before imaging, but for no longer than 1–2 h. Cells and sfGFP localization were visualized by using a Zeiss Axio Imager.Z1 microscope fitted with a 100x EC Plan-Neofluar oil immersion objective (numerical aperture, 1.3). Cells were photographed with an Axiocam MRm cold charge-coupled device camera, using a typical exposure time of 2 s to capture fluorescence images. Live cells were imaged because PBP5 rings were not very sharp in fixed cells. Live cells were imaged in most cases because PBP5 rings were not very sharp in fixed cells. However, the temperature-sensitive strains LP9-1, LP10-1, LMC531 and LMC510 were fixed because live cells started to constrict on the agarose pad while processing the cells for imaging sfGFP-PBP5.

### Immunoblotting

Samples were separated on 12% SDS-PAGE and transferred to nitrocellulose membrane. Membranes were incubated with antibodies at a dilution of 1:10.000 in Tris Buffered Saline containing 50 mM Tris, 150 mM NaCl, 0.05% Tween 20, pH 7.5 (TBST). The secondary antibody was goat anti-rabbit IgG conjugated to horseradish peroxidase (Bio-Rad Laboratories, France) and used at a dilution of 1:10.000 in TBST. Detection was performed with a chemiluminescence plus kit of Amersham Sciences followed by exposure to Hyperfilm (Amersham Biosciences, UK Limited, England).

### PBP5 activity and substrate specificity assays

*In vitro* assays with water-soluble PBP5 (PBP5s) were performed in 20 mM MOPS/NaOH pH 7.0 at 37°C as described (Wilkin *et al*., 1994). The D,D-carboxypeptidase activity of PBP5s (soluble PBP5) on lipid II was tested by using a substrate where the two terminal D-Ala were tritiated. Enzyme (2.6 µg) was incubated for 1 h at 30°C with ^3^H-lipid II in 30 µl 33 mM Tris (pH 7.5) 6.6 mM MgCl_2_ 0.3% decyl-PEG 10% dimethylsulphoxide. The same was done with 4.4 µg of the D,D-carboxypeptidase from *Actinomadura* R39 and ampicillin-inactivated PBP5s. Substrates and products were separated by thin layer chromatography and autoradiographed. The assay showed a clear hydrolysis of D-Ala–D-Ala bond in lipid II by PBP5s. About 80% of the substrate was hydrolyzed by both R39 and PBP5s.

For the *in vitro* assays using membrane-bound PBP5 ^14^C-GlcNAc-labelled lipid II (1.2 nmol, 14 800 dpm) was incubated with 10 mg PBP5 in reaction buffer (10 mM Tris/Maleate; 10 mM MgCl_2_, 50 mM NaCl, 0.2% Triton X-100, pH 6.0) for 1 h at 37°C. A control sample did not contain PBP5. The reaction was stopped by incubation for 15 min at 37°C in the presence of 100 µg ml^−1^ of ampicillin. The samples were then incubated for 1 h at 37°C with 60 µg PBP1B that had been pre-incubated 100 µg ml^−1^ of ampicillin to block its transpeptidation activity. Samples were treated with cellosyl, reduced with sodium borohydrate and analysed by high-performance liquid chromatography (HPLC) as described (Bertsche *et al*., 2005). Peptidoglycan from strain D456 (Edwards and Donachie, 1993) was prepared as described (Glauner, 1988). About 50 mg of peptidoglycan was incubated with 10 µg of PBP5 in reaction buffer for 4 h at 37°C. A control sample did not receive PBP5. The samples were treated with cellosyl, reduced with sodium borohydrate and analysed by HPLC as described (Glauner, 1988). Muropeptides were released from peptidoglycan of strain D456 and were incubated with or without PBP5 prior to reduction and HPLC analysis as described above. Assays with UDP-MurNAc pentapeptide (Kohlrausch and Höltje, 1991) were performed as described for LdcA (Templin *et al*., 1999).

### Peptidoglycan isolation from strain CS703-1 expressing different PBP5 alleles

*Escherichia coli* CS703-1 containing pLP4, pLP514, pLP515 or pLP528 was grown overnight in LB-Kan at 30°C. Next morning, overnight cultures were diluted 1:100 in 250 ml of LB-Kan and incubated at 30°C for 2 h. IPTG was added to a final concentration of 100 µM and incubated for another 3 h. Cells were harvested by centrifugation at 10 000 × *g* for 10 min at 4°C. The cell pellet was resuspended in 6 ml LB and the cell suspension was added to 9 ml of boiling 6% SDS. The samples were incubated in a boiling water bath for 30 min and then stirred overnight at room temperature. Next morning, the samples were centrifuged at 176 000 × *g* for 30 min at 30°C, using a TLA110 rotor in Beckman Optima TLX ultracentrifuge. Samples were washed three times with sterile distilled water and the pellet was resuspended in 1.5 ml of 10 mM Tris-HCl (pH 7.0). α-Amylase was added to a final concentration of 100 µg ml^−1^ and incubated at 37°C for 2 h, after which pronase was added to a final concentration of 100 µg ml^−1^ and the samples were incubated at 60°C for 2 h. Pronase activity was destroyed by boiling the samples in 0.5% SDS for 30 min. Samples were washed three times with sterile distilled water. Muropeptides were released by cellosyl (kindly provided by Hoechst, Frankfurt, Germany) and reduced with sodium borohydride prior to HPLC analysis according to a published procedure (Glauner, 1988).
