# Supplementary material for: Septal and lateral wall localization of PBP5, the major D,D-carboxypeptidase of Escherichia coli, requires substrate recognition and membrane attachment
Source: Mol Microbiol. 2010 Jun 7;77(2):300–23. doi: 10.1111/j.1365-2958.2010.07205.x (PMC2909392; doi:10.1111/j.1365-2958.2010.07205.x)
Supplement: Supplementary file 1 [file mmi0077-0300-SD1.pdf]

D,D-carboxypeptidase of *Escherichia coli,* requires substrate recognition and membrane attachment

Lakshmiprasad Potluri[4], Aneta Karczmarek[1,] Jolanda Verheul[1], Andre Piette[2], Jean-Marc Wilkin[2§], Nadine Werth[5], Manuel Banzhaf[3], Waldemar Vollmer[3], Kevin D. Young[4], Martine Nguyen-Distèche[2], and Tanneke den Blaauwen[1*]

Supplementary discussion

Cross-reactivity of anti-PBP5 and localization of PBP6

Because PBP5 and PBP6 from *E. coli* are so similar, one would expect that their cellular localization might be similar, as well. The crystal structure of PBP5 consists of two modules orientated at an angle of 90° to one another (Davies et al., 2001, Nicholas et al., 2003). The core enzymatic domain is conserved among PBP5, PBP6, and PBP6B (DacD) and probably contains the epitopes that are recognized by a large fraction of IgG molecules in PBP5 antiserum, and which therefore cross-react with PBP6. The second PBP5 module is β-sheet rich and contains the carboxy-terminal membrane associated amphipathic helix. This domain is much less conserved (Nelson & Young, 2001) and may allow selection of a subset of IgG molecules that can discriminate between PBPs 5 and 6. Although our affinity purified antiserum clearly cross-reacted with PBP6 as determined by immunoblot analysis, we were able to purify the antiserum further by absorbing cross-reacting species to a cell extract derived from an *E. coli* strain lacking PBP5. However, whether we used this PBP5-specific antiserum or the original cross-reacting antiserum to visualize the cellular distribution of the PBPs, the pattern of PBP5 localization was the same. Since the cross-reacting antibody would also be expected to bind to PBP6, the results indicate either that PBPs 5 and 6 have exactly the same localization pattern and the same substrate

dependence for localization, or else that PBP6 is not expressed at high levels in logarithmic phase wild type cells.

Supplementary Tables

Table S1. Properties of purified soluble PBP5

Properties	Parameters	PBP5
Absorption U.V.	λ_{max} (nm)	278 ± 2
	$\Delta\varepsilon_{280}$($M^{-1}.cm^{-1}$)	46300 ± 400
Fluorescence	λ_{max} (nm) (native)	342 ± 2
	λ_{max} (nm) (denatured)	354 ± 2
C.D.	Minimum (nm)	208.5 ± 0.5
Charge	pI	7.8 ± 0.3
Stability	Tm (°C)	52.6 ± 0.3
	Reversibility	no

Table S2. Transpeptidation /hydrolysis (T/H) ratio for the purified water soluble PBP5 was analyzed with $CO-C_6H_5-NH-CH_2-CO-S-CH_2-COO^-$ (S2a) as donor and various amino acids as acceptor

Acceptor	T/H[a]
D-alanine	2.7
D-lactate	0.5
A2pm	0.32
D-leucine	1.5
D-histidine	1.5

D-phenylalanine	2.7
glycylglycine	0.3
D-serine	3.0
D-valine	0.3
D-asparagine	0.4

[a]T/H (transpeptidation/hydrolysis) ratio was determined after complete utilisation of the donor substrate S2a (250 µM) in the presence of 20 mM acceptor with the exception of D-Phe and A2pm where 10 mM was used. Standard deviation values are 15 % or less.

Supplementary figures

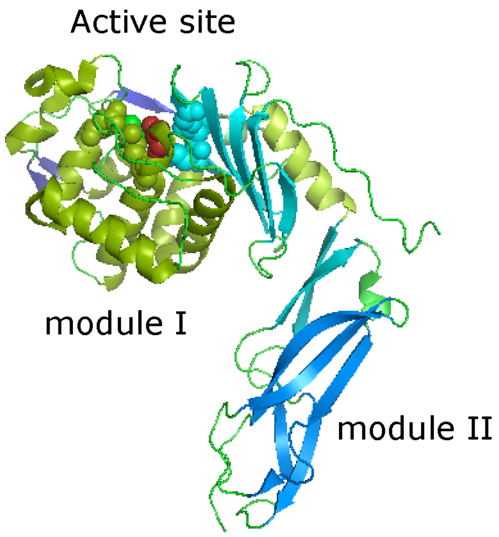

Fig. S1. Structure of *E. coli* PBP5 (Nicholas et al., 2003). The conserved active site residues, responsible for the the D,D-carboxypeptidase activity, are present in module I and are represented as spheres. The red sphere represents Serine44

of the active site, which was mutated to glycine in this study. Helices are in green shades and strands in blue shades. Module II is connected to the periplasmic side of the cytoplasmic membrane by an amphiphathic helix at the carboxy-terminus (O'Daniel et al., 2010). This helix is not included in the crystal structure.

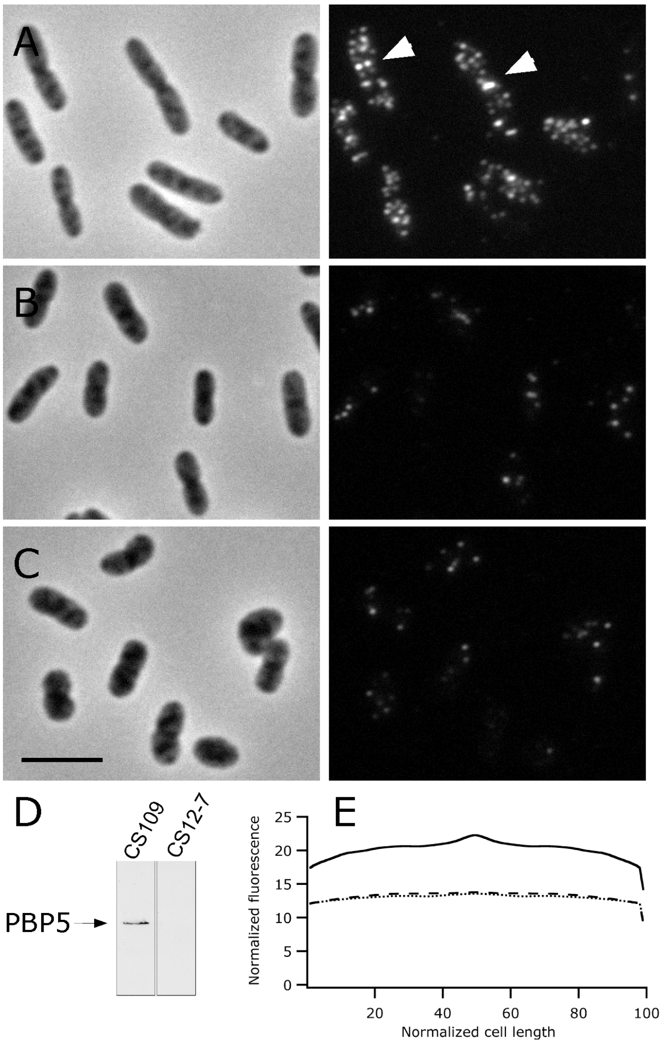

Fig. S2. The affinity-purified antiserum against PBP5 is specific for PBP5. A. Immunolabelling of PBP5 in the wild type strain CS109 (the parent of CS12-7) shows a foci-like lateral fluorescence pattern and midcell localization (see arrows). B. No localization is observed in the PBP5 deletion strain CS12-7 or (C) in the strain CS703-1 that lacks the major D,D-carboxypeptidases PBP4, PBP5 and PBP6. The foci visible in the latter two strains are also observed even when the cells are labelled only with secondary antibodies. The bar equals 5 μm. Phase contrast images are on the left and fluorescence images are on the right. D.

Immunoblot of a cell extract of the PBP5 deletion strain CS12-7 (Δ*dacA*) right lane and its parental strain CS109 (left lane). A single band at approximately 40 kDa corresponds to the molecular mass of PBP5. E. Normalized average fluorescence intensity profile of the wild type strain CS109 (solid line) and the two PBP5 deletion strains CS12-7 (dotted line) and CS703-01 (dashed line) plotted against the normalized cell length of the cells (N > 500 cells).

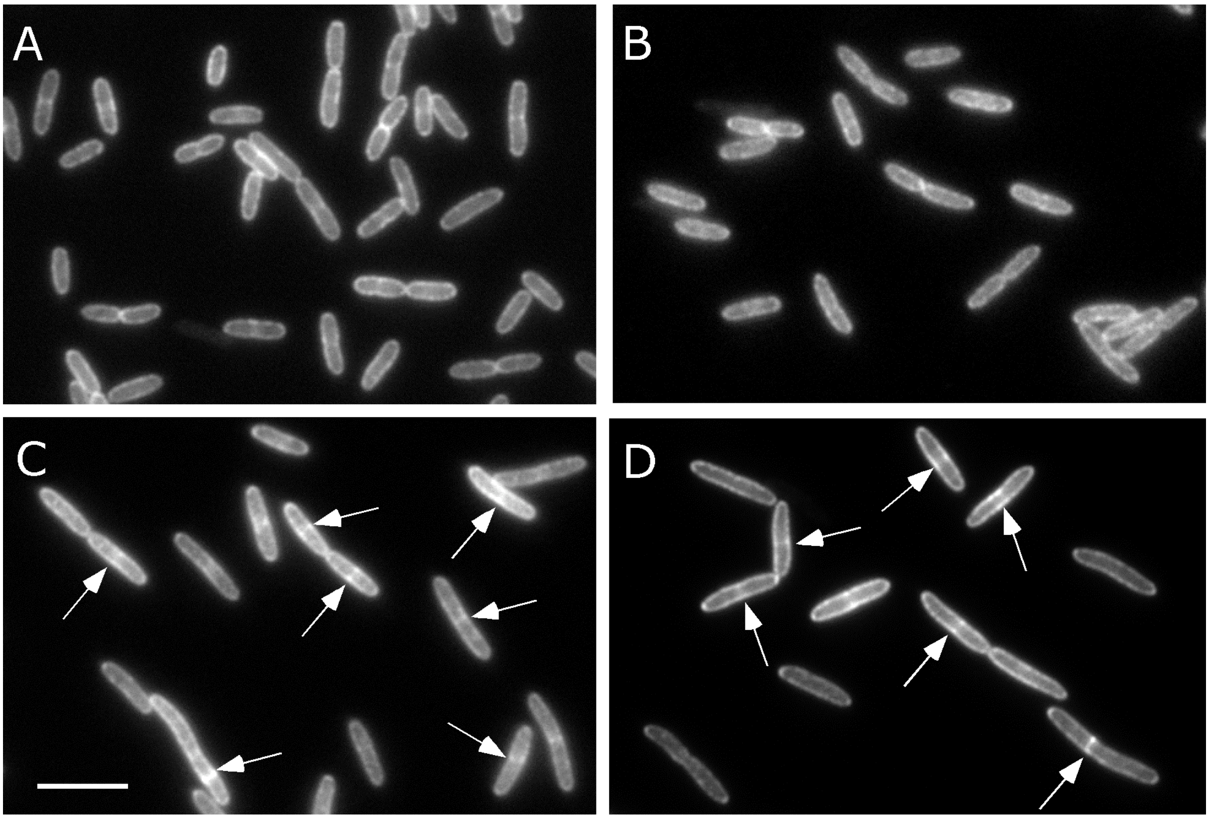

Fig. S3. Strain LMC531pLP521 and LMC510pLP521 expressing DbsA(ss)-sfGFP-PBP5 grown in GB1 in the presence of 50 μM IPTG for 2 MDs at 28°C (A and B, respectively) or at 42°C (C and D, respectively). The arrows indicate bands of sfGFP-PBP5 localization perpendicular to the length axis. The bar equals 5 μm.

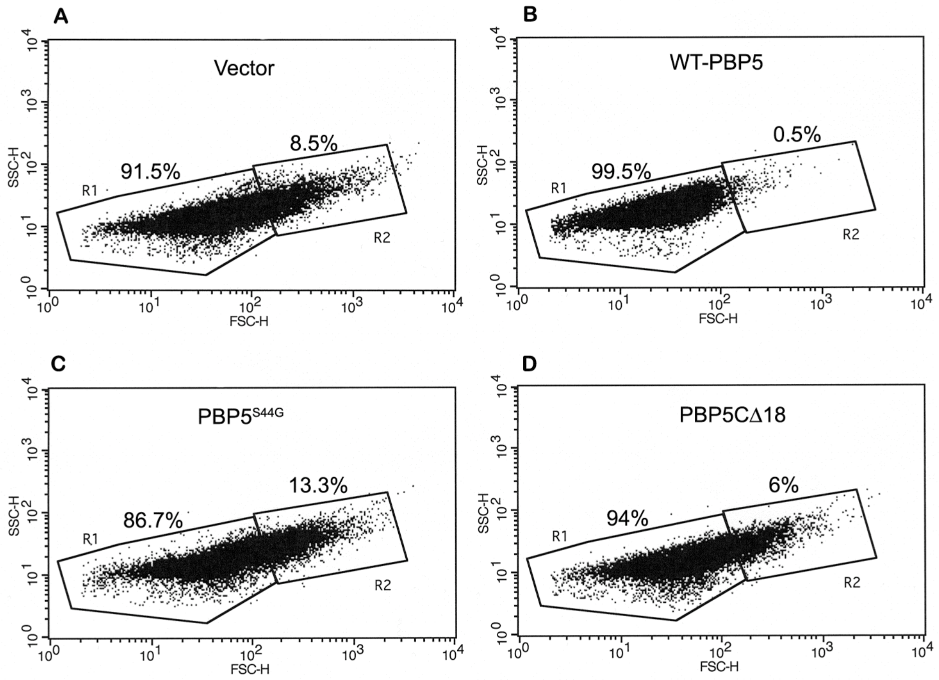

Fig. S4. Tail-less PBP 5 weakly complements the cell shape defects of *E. coli* CS703-1, which lacks the three major D,D-carboxypeptidases .

E. coli strain CS703-1 containing plasmids pLP9 expressing DbsA(ss)-sfGFP (A), pLP515 expressing DbsA(ss)-sfGFP-WT-PBP5 (B), pLP514 expressing DbsA(ss)-sfGFP-PBP5(S44G) (C) or pLP528 expressing DbsA(ss)-sfGFP-PBP5CΔ18 (D) were grown in LB at 30°C and their cell shape was analyzed with a FACS machine, as described in the experimental procedures. Each panel represents the cell shape distribution of the respective strain on a dot plot; obtained by measuring the forward-scattered light (FSC - *x* axis) versus side-scattered light (SSC - *y* axis). Region 1 (R1) represents the cells with less complex shapes and region 2 (R2) represents the cells with more complex shapes.

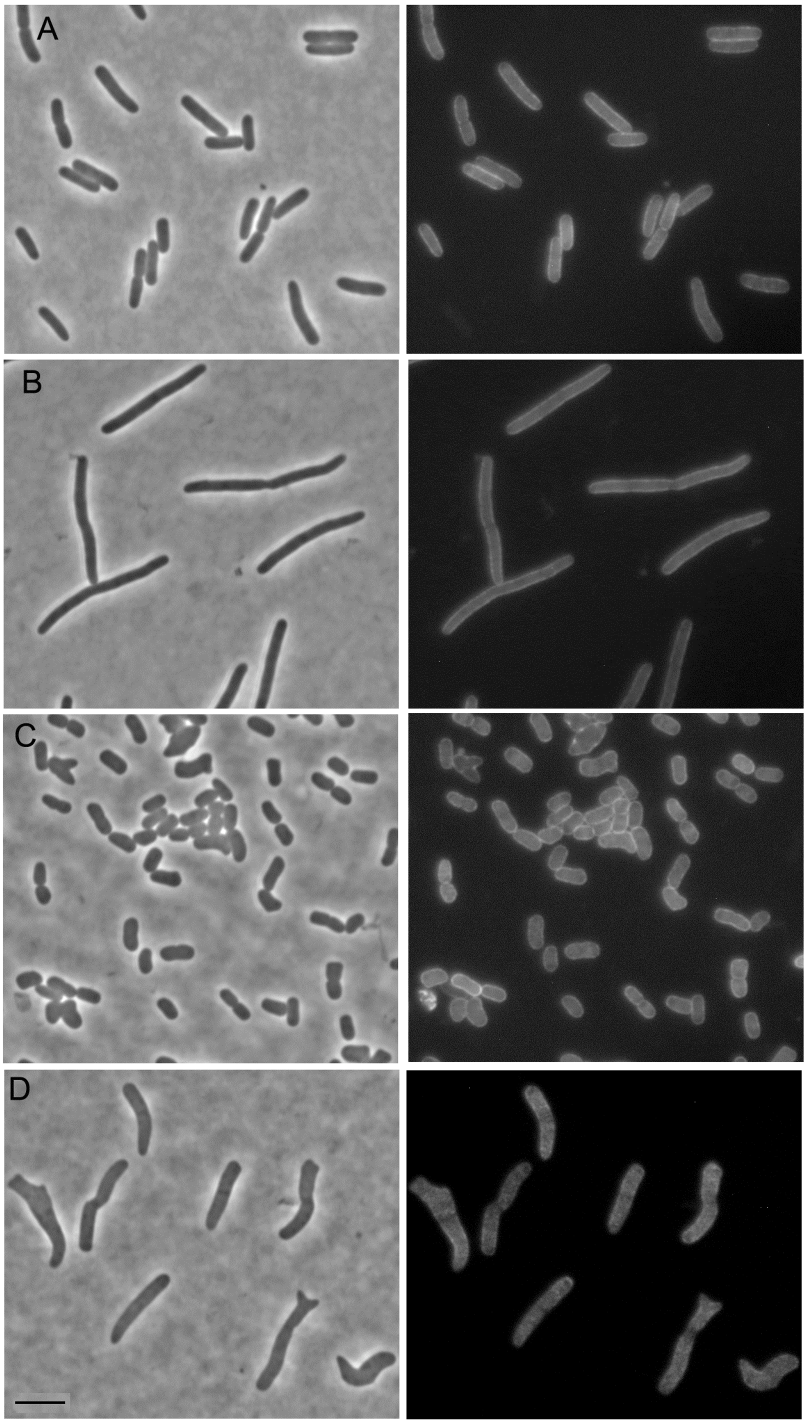

Fig. S5. PBP5S44G lacking the C-terminal amphipathic helix avoids the septum. (A) Wild type strain, CS109, expressing DsbA(SS)-sfGFP-PBP5(S44G)CΔ18 fusion protein from plasmid pLP524. (B) CS109 strain expressing DsbA(SS)-sfGFP-PBP5(S44G)CΔ18 fusion protein were grown in LB at 30°C and in the presence of 1 aztreonam for 1 MD. (C) CS703-1, that lacks the three major D,D-carboxypeptidases, expressing DsbA(SS)-sfGFP-PBP5(S44G)CΔ18 fusion protein

from plasmid pLP524. (D) CS703-1 strain expressing DsbA(SS)-sfGFP-PBP5(S44G)CΔ18 fusion protein in the presence of aztreonam for 1 MD. The left side of each dual panel is the phase contrast image and the right side is the fluorescence image. All images have the same magnification, and the scale bar in panel D equals 5 μm.

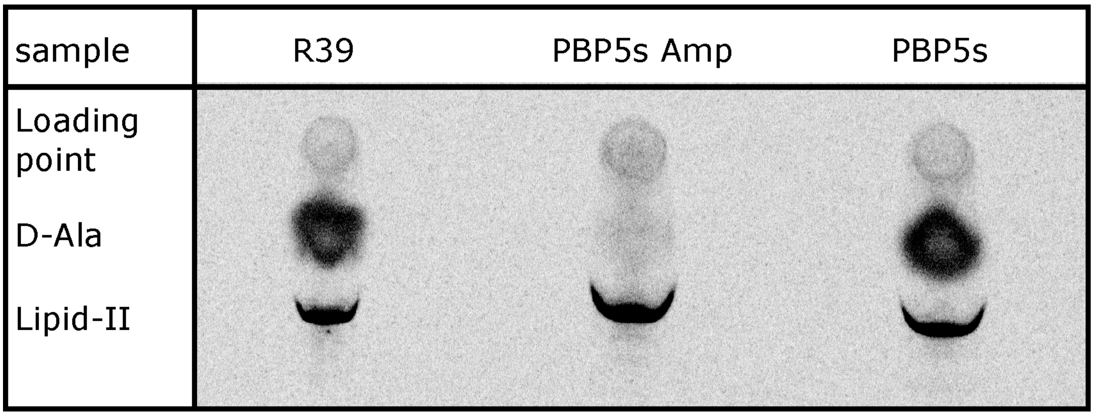

Fig. S6. Autoradiograph of thin layer chromatograph of ^{3}H-lipid II (where the two terminal D-Ala were tritiated) incubated for one hour at 30°C with 4.4 μg of the D,D-carboxypeptidase from *Actinomadura* R39, 2.6 μg ampicillin-inactivated soluble PBP5 and 2.6 μg soluble PBP5. The assay showed a clear hydrolysis of D-Ala–D-Ala bond in lipid II by PBP5s. About 80 % of the substrate was hydrolyzed by both R39 and PBP5s.

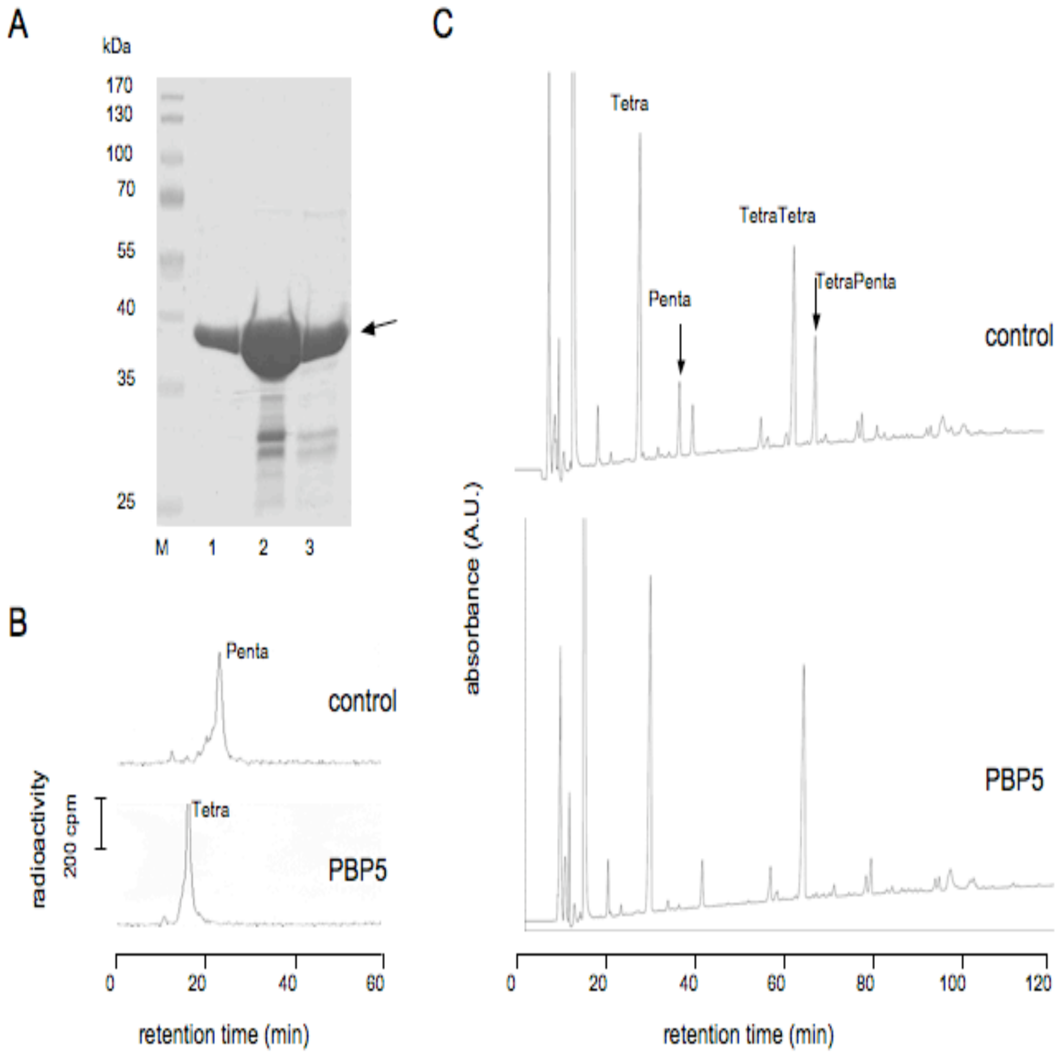

Fig. S7. A. Fractions from cation exchange chromatography containing purified PBP5 (arrow). B. Activity of PBP5 against lipid II. C. Activity of PBP5 against pentapeptides present in peptidoglycan from strain D456. Tetra, disaccharide tetrapeptide; penta, disaccharide pentapeptide; TetraTetra, bis-disaccharide tetratetrapeptide; TetraPenta, bis-disaccharide tetrapentapeptide.
